# Diagnostic accuracy of natriuretic peptide screening for left ventricular systolic dysfunction in the community: systematic review and meta‐analysis

**DOI:** 10.1002/ehf2.14314

**Published:** 2023-02-13

**Authors:** Clare R. Goyder, Andrea K. Roalfe, Nicholas R. Jones, Kathy S. Taylor, Charles D. Plumptre, Olivia James, Thomas R. Fanshawe, F D Richard Hobbs, Clare J. Taylor

**Affiliations:** ^1^ Nuffield Department of Primary Care Health Sciences University of Oxford Oxford UK; ^2^ Nuffield Department of Medicine University of Oxford Oxford UK; ^3^ Clinical Medical School, University of Oxford, Level 3 John Radcliffe Hospital Oxford UK

**Keywords:** Biomarkers, Screening, Left ventricular systolic dysfunction, Natriuretic peptides

## Abstract

**Aims:**

Heart failure (HF) is a global health burden and new strategies to achieve timely diagnosis and early intervention are urgently needed. Natriuretic peptide (NP) testing can be used to screen for left ventricular systolic dysfunction (LVSD), but evidence on test performance is mixed, and international HF guidelines differ in their recommendations. Our aim was to summarize the evidence on diagnostic accuracy of NP screening for LVSD in general and high‐risk community populations and estimate optimal screening thresholds.

**Methods:**

We searched relevant databases up to August 2020 for studies with a screened community population of over 100 adults reporting NP performance to diagnose LVSD. Study inclusion, quality assessment, and data extraction were conducted independently and in duplicate. Diagnostic test meta‐analysis used hierarchical summary receiver operating characteristic curves to obtain estimates of pooled accuracy to detect LVSD, with optimal thresholds obtained to maximize the sum of sensitivity and specificity.

**Results:**

Twenty‐four studies were identified, involving 26 565 participants: eight studies in high‐risk populations (at least one cardiovascular risk factor), 12 studies in general populations, and four in both high‐risk and general populations combined. For detecting LVSD in screened high‐risk populations with N‐terminal prohormone brain natriuretic peptide (NT‐proBNP), the pooled sensitivity was 0.87 [95% confidence interval (CI) 0.73–0.94] and specificity 0.84 (95% CI 0.55–0.96); for BNP, sensitivity was 0.75 (95% CI 0.65–0.83) and specificity 0.78 (95% CI 0.72–0.84). Heterogeneity between studies was high with variations in positivity threshold. Due to a paucity of high‐risk studies that assessed NP performance at multiple thresholds, it was not possible to calculate optimal thresholds for LVSD screening in high‐risk populations alone. To provide an indication of where the positivity threshold might lie, the pooled accuracy for LVSD screening in high‐risk and general community populations were combined and gave an optimal cut‐off of 311 pg/mL [sensitivity 0.74 (95% CI 0.53–0.88), specificity 0.85 (95% CI 0.68–0.93)] for NT‐proBNP and 49 pg/mL [sensitivity 0.68 (95% CI 0.45–0.85), specificity 0.81 (0.67–0.90)] for BNP.

**Conclusions:**

Our findings suggest that in high‐risk community populations NP screening may accurately detect LVSD, potentially providing an important opportunity for diagnosis and early intervention. Our study highlights an urgent need for further prospective studies, as well as an individual participant data meta‐analysis, to more precisely evaluate diagnostic accuracy and identify optimal screening thresholds in specifically defined community‐based populations to inform future guideline recommendations.

## Introduction

Approximately 40 million people worldwide are living with heart failure (HF), representing a major public health burden,^1^, ^2^ but treatment can improve quality of life and survival and reduce hospitalizations.^3^, ^4^ Detecting HF in the community, especially in the early stages, to achieve timely diagnosis is an urgent research priority.^5^, ^6^, ^7^ There is reliable evidence that treatment with renin–angiotensin–aldosterone system (RAAS) inhibitors, titrated to the appropriate dose, can limit progression from left ventricular systolic dysfunction (LVSD) to HF.^8^, ^9^, ^10^, ^11^ Screening is one potential route to detect LVSD and provides an important opportunity for early intervention.

Echocardiography, and more recently cardiac magnetic resonance imaging (MRI), can accurately identify patients with LVSD, but these strategies are not feasible as general population screening tools due to poor cost‐effectiveness. One potential screening strategy is an initial natriuretic peptide (NP) blood test with subsequent echocardiography or MRI performed only in patients with raised NP levels. There is evidence supporting the role of NP testing in the diagnosis of HF in symptomatic patients and limited evidence in treatment optimization.^12^, ^13^ However, the St Vincent's Screening to Prevent Heart Failure (STOP‐HF) trial was the first randomized controlled trial to indicate that an NP‐guided screening approach in asymptomatic people, linked to targeted prevention, reduced the progression of asymptomatic LVSD and the development of HF in high‐risk groups.^13^

Globally, HF guidelines differ in their recommendations on NP screening to detect LVSD: European guidelines do not currently advocate screening, whereas North American guidelines recommend NP screening in high‐risk patients but do not specify optimal NP screening thresholds or make any recommendation for general populations.^4^, ^14^, ^15^, ^16^, ^17^ Moreover, the overall performance of NP as a screening tool for LVSD remains unclear. How NP performs as a screening test across different populations (e.g. high‐risk vs. general community populations) and what NP threshold is appropriate for biomarker‐based screening is also uncertain. Previous systematic reviews have focused on the accuracy of NP as a diagnostic tool for HF in symptomatic, presenting patients,^17^, ^18^ or analysed accuracy in combined diagnostic and screening studies.^18^, ^19^ Those reviews that have assessed NP performance in a screening context have often combined primary and secondary care studies, rather than focussing specifically on screening in the community.^18^, ^20^, ^21^

The aim of this study was to provide an up‐to‐date summary of the accuracy of NP screening for LVSD, considering both brain natriuretic peptide (BNP) and N‐terminal prohormone BNP (NT‐proBNP) compared with echocardiography and cardiac MRI in community populations, and to determine the optimal NP screening threshold.

## Methods

This systematic review and meta‐analysis protocol has been prospectively registered in PROSPERO (registration number: CRD42018087498) and separately published.^22^ This review has been produced in accordance with the Preferred Reporting Items for Systematic Reviews and Meta‐analyses (PRISMA) guidelines and recommendations from the Cochrane Collaboration.^23^ The full search strategy is available in *Appendix* *S1*.

This study was discussed with our patient and public involvement group, who agreed that improving the detection of HF was a research priority. The group was familiar with the consequences of delayed diagnosis, some with personal experience, and welcomed more research on screening.

### Search methodology

We searched Ovid Medline, Embase, Cochrane Database of Systematic Reviews and Cochrane Central Register of Controlled Trials, Cochrane CENTRAL, DARE, and Science Citation Index from inception on 17 April 2019 and updated the search on 13 August 2020. We did not employ a study design filter or add any language restriction. We searched reference lists to identify more publications. Screening was carried out by two reviewers independently (CG and either CP, NJ, or OJ). Disagreements were resolved by discussion or referral to a third reviewer (CT).

### Screening methodology

We included studies of over 100 adult participants in a community setting to limit bias from small studies. Included studies compared the performance of NP testing with either echocardiography or cardiac MRI for the detection of LVSD. We included studies that recruited screened community populations. Community screening may identify patients with preclinical HF, such as asymptomatic LVSD, also termed stage B HF. Screening may also detect patients with clinical HF who had not been appropriately diagnosed previously. Given this, we took a pragmatic approach and included all patients who had participated in screening studies.

We excluded studies of patients recruited through secondary care, such as cardiology clinics, and studies presenting insufficient data to construct 2 × 2 tables. We also excluded duplicate datasets, selecting the papers that most closely aligned with inclusion criteria or were most recently published. The inclusion and exclusion criteria for participants in included studies are found in *eTable* *1*.

### Target condition

Consistent with the overall aim of community screening, LVSD was defined broadly by evidence of reduced ejection fraction (EF) including either quantitative or narrative descriptions of reduced systolic function and/or other echocardiographic parameters. An EF cut‐off of 40% (or nearest) was selected in studies reporting results for more than one EF threshold.

### Data extraction and quality assessment

Data extraction was performed independently by two reviewers (CG and either CP, NJ, or OJ).^22^ A risk of bias template incorporating QUADAS‐2 criteria was used to assess methodological quality.^24^ Disagreements were discussed or referred to a third reviewer (CT). Diagnostic accuracy data were extracted from all studies,^25^ and 2 × 2 tables were constructed in accordance with current reporting guidelines, at all NP thresholds reported.^26^

### Statistical analyses

Meta‐analysis was performed where there were at least four studies with available data. Subgroup analyses were performed for both types of NP (to include BNP and NT‐proBNP) as well as high‐risk and general populations. High‐risk populations were defined as having at least one cardiovascular (CV) risk factor or ischaemic heart disease (IHD) or were selected non‐general populations, such as cohorts of nursing home residents or patients with chronic obstructive pulmonary disease (COPD), as the overlap between COPD and HF is known to be high.^27^

To visually explore the variation in diagnostic accuracy, sensitivity and specificity forest plots with 95% confidence intervals (CIs) for both types of NP, ordered by each threshold reported, were produced in RevMan 5.3.^28^ Where data included multiple thresholds, R version 3.5.3 (*diagmeta* package)^29^ was used to produce SROC curves in relation to the positivity threshold with estimation of the single threshold that maximized the sum of sensitivity and specificity.^30^ A logistic distribution for threshold within the diseased and non‐diseased groups was assumed, and NP levels were log‐transformed due to skewness. If there were insufficient data to generate SROCs with multiple thresholds, hierarchical SROC curves with 95% CI and prediction regions were drawn (Stata version 15.0, *metandi* command) using the lowest threshold for each study.^31^ Some studies did not report data for the combined group of men and women together with the threshold used to define positivity. Therefore, for our primary analysis, we decided to include all the available data for men and totals (i.e. men and women combined if reported, and otherwise men only). Sensitivity analysis was carried out to analyse the available data on women and totals (i.e. men and women combined if reported, and otherwise women only) to explore whether there were any differences that resulted from this analysis decision. Sensitivity analyses were also carried out to compare studies that excluded participants with a previous diagnosis of LVSD with studies that did not and to examine whether there was any difference in accuracy in studies that described participants as entirely asymptomatic compared with other included studies.

## Results

**Figure 1:**
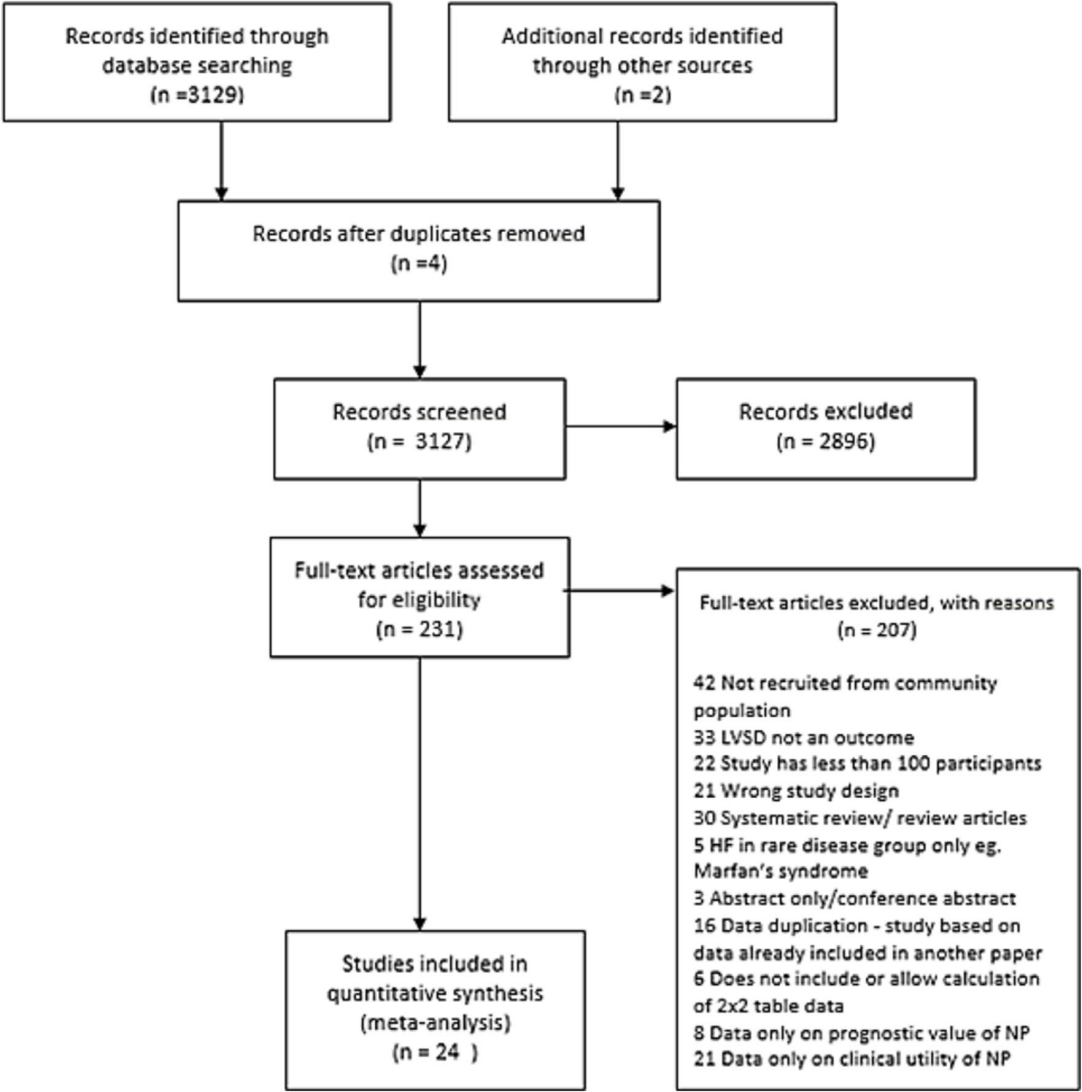
PRISMA diagram flow of studies through the selection process.

From 3131 records (*Figure* *1*), 24 studies presented accuracy data for NP screening to detect LVSD, involving 26 565 participants^32^, ^33^, ^34^, ^35^, ^36^, ^37^, ^38^, ^39^, ^40^, ^41^, ^42^, ^43^, ^44^, ^45^, ^46^, ^47^, ^48^, ^49^, ^50^, ^51^, ^52^, ^53^, ^54^, ^55^; all included studies were cross‐sectional. The included studies, with population characteristics and the lowest threshold reported, are summarized in *Table* *1*. Full data for all thresholds including prevalence are available in *eTable* *2*. Details of inclusion and exclusion criteria are listed in *eTable* *1*; included studies were published over 16 years between 1998 and 2014.

**Table 1:** Characteristics of included studies reporting NP screening for LVSD.

Studies	Location	Natriuretic peptide	Number of participants	Age group(yrs)	Population subgroup	Lowest LVSD threshold(pg/mL)	Target condition
Abhayaratna 2006§	Canberra, Australia	NT‐proBNP	1229	60–74	General population⁂	151	EF ≤ 40% and advanced DD‐NEF║
Betti 2009§	Unclear, Italy	NT‐proBNP	1012	> = 67	High‐risk⁂	125	Pooled systolic and moderate–severe DD¶
Cosin Aguilar 2003	Valencia, Spain	NT‐proBNP	203	49–81	General population	215	LVEF ≤50%
Costello‐Boerrigter 2006	Olmsted County, Minnesota, USA	BNP	1869	<65	General population⁂	12	LVEF ≤40% /LVEF ≤50%
		NT‐proBNP	1869	<65	General population⁂	32	
De Lemos 2009	Dallas, Texas, USA	BNP	2429	18–64	General population⁂	7.1	LVSD EF < 55 or LVH††
		NT‐proBNP	2429	18–64	General population⁂	28.9	
Galasko 2005	London, UK	NT‐proBNP	734	> = 45	General population	†	LVEF <50%
		NT‐proBNP	761	> = 45	High‐risk	†	LVEF<40%
Gavazzi 2014	Lombardy, Italy	NT‐proBNP	623	55–80	High‐risk	†	LVEF <40% LVEF <45% LVEF <50%
Goetze 2006	Copenhagen, Denmark	NT‐proBNP	3497	>55	General population⁂	144	LVEF <40%
Groenning 2004	Copenhagen, Denmark	NT‐proBNP	672	> = 50	General population	351	LVSD LVEF ≤35% LVSD LVEF <40% LVSD LVEF <45% LVSD LVEF <50%
Hebert 2010	Louisiana, USA	BNP	145	40–75	High risk	60	LVEF < = 54%
Hedberg 2004	Västerås, Sweden	BNP	407	75	General population	28	LVSD based on wall motion index corresponding to LVEF <40%
Hobbs 2004	England, UK	BNP	133	> = 45	High risk	103.8	LVSD<40%
		BNP	307	> = 45	General population	103.8	LVSD<40%
		NT‐proBNP	133	> = 45	High risk	338	LVSD <40%
		NT‐proBNP	307	> = 45	General population	338	LVSD <40%
Lobos Bejarano 2012	Madrid, Spain	BNP	204	>63	High risk	71	LVSD EF < 50%
Luchner 2000	Augsburg, Germany	BNP	672	50–67	General population	34	LVD (defined as fractional shortening <28%)/general population
Luers 2010	Goettingen, Germany	BNP	542	>40	High risk	105	LVSD (LVEF <50%)
Lukowicz 2005	Augsburg, Germany	BNP	1678	25–75	General population	27	LVSD (EF < 40%)
Mason 2013	North‐East England, UK	BNP	392	≥ 65	High risk	145	LVSD EF < 50%
		NT‐proBNP	393	≥ 65	High risk	1000	
McDonagh 1998	Glasgow, UK	BNP	1252	25–74	General population	17.9	LVSD <30%
		BNP	1252	25–74	High risk	17.9	
Mureddu 2013	Lazio region, Italy	NT‐proBNP	435	65–84	High risk	278	LVSD EF < 50
		NT‐proBNP	1452	65–84	General population	278	
Murtagh 2012	Ireland	BNP	814	>40	High‐risk	20	LVSD <50% LVSD <40%
Ng 2003	Leicestershire, UK	BNP	1331	45–80	General population	66	Definite systolic EF ≤ 35%
		NT‐proBNP	1331	45–80	General population	318	
Smith 2000	Poole, Dorset, UK	BNP	155	>70	General population	64.7	Reduced left ventricular ejection fraction/LVSD confirmed by echo
Vasan 2002	Framingham, Massachusetts, USA	BNP	3177	>40	General population⁂	21	

Population subgroup describes total (men plus women) except for ⁂ which describes only men *Galasko 2005 thresholds based on age/sex specific 97.5th percentiles: 100 pg/mL males aged 45–59; 164 pg/mL females aged 45–59; 172 pg/mL males aged > = 60; 225 pg/mL females age > = 60 †Gavazzi 2014 thresholds based on age/sex specific 95th percentiles (data not provided)§. LVSD data pooled with data on diastolic dysfunction. ║DD‐NEF diastolic dysfunction LV EF (EF > 50%), ¶DD diastolic dysfunction ††Left ventricular hypertrophy. The threshold shown in this table is the lowest threshold reported; all threshold data are reported in *eTable* *2*.

### Characteristics of included studies

Eight studies included data on only high‐risk populations,^32^, ^33^, ^34^, ^35^, ^36^, ^37^, ^38^, ^39^ 12 included only general populations,^37^, ^40^, ^41^, ^42^, ^43^, ^44^, ^45^, ^46^, ^47^, ^48^, ^49^, ^50^ and four included data on high‐risk and general populations.^51^, ^52^, ^53^, ^54^ Nine studies assessed NT‐proBNP only,^32^, ^33^, ^34^, ^40^, ^43^, ^46^, ^47^, ^51^, ^54^ and nine studies assessed BNP only; one of these used a point‐of‐care BNP test, Biosite®,^35^ whereas all other tests were processed in a laboratory. Six studies evaluated performance in both biomarkers.^37^, ^39^, ^42^, ^43^, ^48^, ^52^ All studies used echocardiography as the comparator except one that used cardiac MRI.^45^ Subgroup sample sizes corresponding to each meta‐analysis were smaller for high‐risk populations with NT‐proBNP (*n* = 4193) and BNP (*n* = 2940), with larger groups seen in the general populations, with NT‐proBNP (*n* = 13 416) and BNP (*n* = 12 970).

Studies differed in the inclusion criteria for the ages of participants they recruited. General populations included participants of younger ages, whereas high‐risk populations were older (as summarized in *Table* *1*).

Most included studies classified LVSD as reduced EF of either <40% or <50%. In two studies, the outcomes of diastolic dysfunction and LVSD were combined,^32^, ^40^ and three studies used narrative descriptions of LVSD rather than quantitative measurements of EF.^32^, ^41^, ^42^

The reported prevalence for studies presenting accuracy data for LVSD in total populations ranged from 0.4% to 12.9% (*eTable* *2*).

There was some variability in how individual studies defined high‐risk populations. Some studies categorised any participant who had more than one CV risk factor as high risk.^37^, ^38^ One only included patients who had IHD in a high‐risk group,^53^ whereas another included post‐myocardial infarction (MI) or IHD patients in combination with other risk factors.^47^ There were no included studies that included cohorts of COPD patients; one high‐risk study recruited from nursing homes.^39^

Only seven studies described the population as completely asymptomatic.^32^, ^33^, ^36^, ^37^, ^38^, ^42^, ^54^ One study described the population as ‘mostly asymptomatic’.^55^ McDonagh et al. described the population as 50% symptomatic and 50% asymptomatic.^53^ Of the remaining studies, the majority recruited randomly selected community populations and did not provide details of whether the participants reported any symptoms. Some described their population cohorts as ‘healthy’ without any data to explain this.^45^ Two studies described participants as having a high proportion of symptomatic participants^35^, ^39^ and one study recording only 22.2% as symptomatic.^43^

### Quality of included studies

The majority of studies had low or unclear risk of bias with <20% of ratings in the high‐risk category (*eFigure* *1*, *eFigure* *2*). Studies recorded with high risk for patient selection generally excluded patients who had a previous MI even though this patient group may benefit from screening for LVSD.^32^, ^42^, ^45^ In reporting the index test, some studies presented incomplete data.^44^, ^49^, ^55^ Not all studies performed the reference test blinded to the index test,^55^ and some failed to give information on how echocardiography was conducted.^53^

### Accuracy of NP to screen for LVSD in high‐risk populations

**Figure 2:**
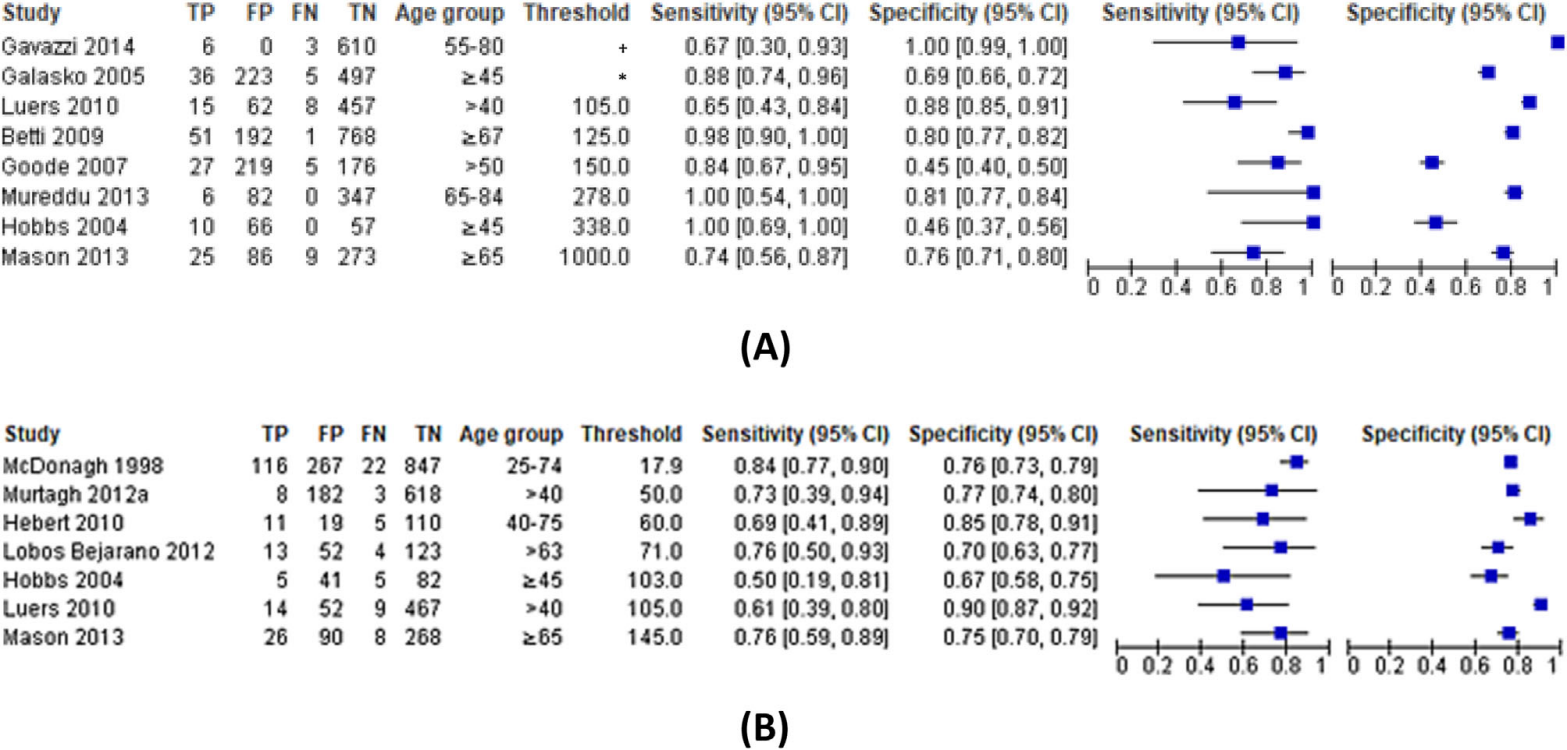
(A) Paired sensitivity and specificity plot for NT‐proBNP compared with echocardiography for detecting LVSD in screened high‐risk populations. Thresholds are measured in pg/mL, and studies are ordered by NP threshold. Threshold were age specific for Gavazzi et al., based on 95th percentiles (data not provided) and for Galasko et al*.*, based on 97.25th percentiles (100 pg/mL males aged 45–59; 164 pg/mL females aged 45–59; 172 pg/mL males aged > = 60; 225 pg/mL females age> = 60). (B) Paired sensitivity and specificity plot for BNP compared with echocardiography for detecting LVSD in screened high‐risk populations. Thresholds are measured in pg/mL, and studies are ordered by NP threshold.

**Figure 3:**
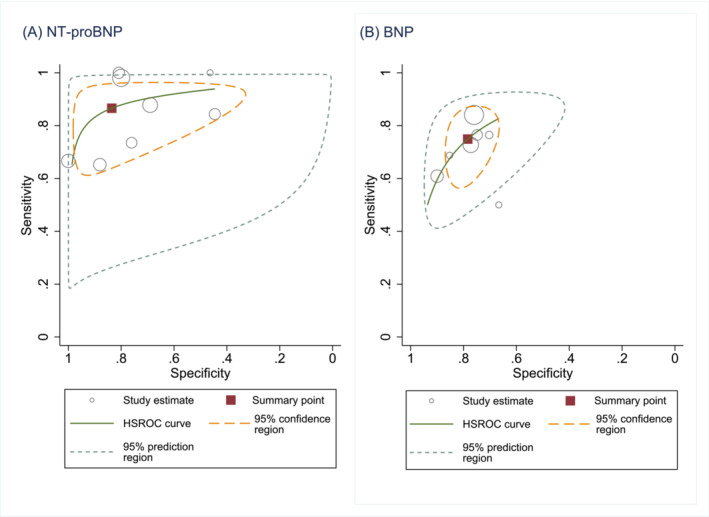
(A) SROC curves of NT‐proBNP compared with echocardiography for detecting LVSD in screened high‐risk populations. (B) SROC curves of BNP compared with echocardiography for detecting LVSD in screened high‐risk populations.

Most studies of high‐risk populations demonstrated a trade‐off between sensitivity and specificity as shown in the forest plots for NT‐proBNP and BNP (*Figure* *2* *A*). For NT‐proBNP (*Figure* *3* *A*), the pooled sensitivity was 0.87 (95% CI 0.73–0.94) and specificity 0.84 (95% CI 0.55–0.96) for detecting LVSD in screened high‐risk populations. For BNP in high‐risk populations (*Figures* *2* *B* *and* *3* *B*) the pooled sensitivity was 0.75 (95% CI 0.65–0.83) and specificity 0.78 (95% CI 0.72–0.84).

### Accuracy of NP to screen for LVSD in general populations

There was extensive variability in sensitivity and specificity for studies of general populations (*eFigure* *3*), some of which was due to differences in reported threshold. For NT‐proBNP in general populations (*eFigure* *4*), the pooled sensitivity was 0.72 (95% CI 0.42–0.90) with specificity 0.82 (95% CI 0.60–0.93), and the optimal threshold was 274 pg/mL. For BNP in general populations, the pooled sensitivity was 0.62 (95% CI 0.32–0.85) with specificity 0.83 (95% CI 0.61–0.94) and optimal threshold 46 pg/mL (*eFigure* *1*).

### Optimal screening thresholds

**Figure 4:**
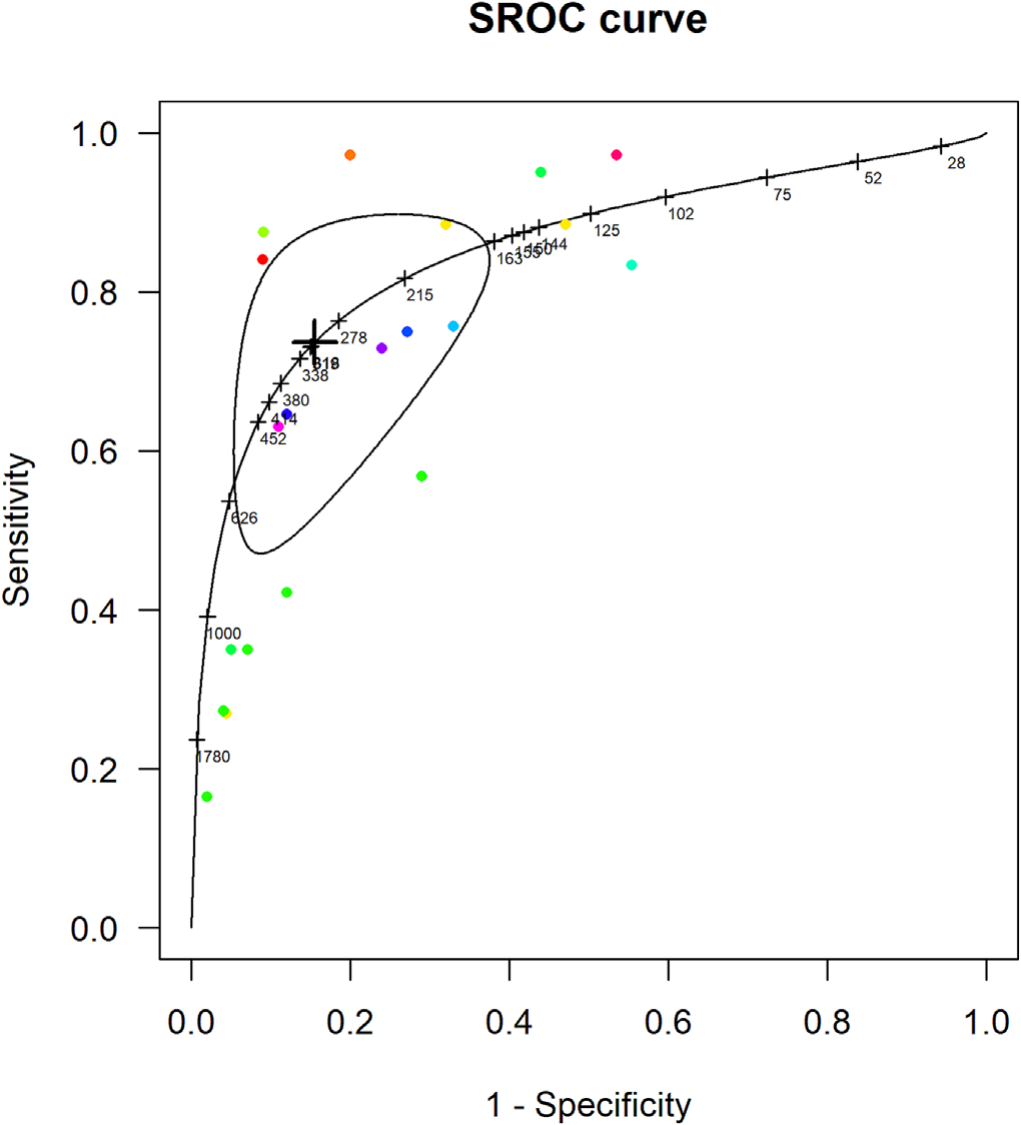
SROC curve of NT‐proBNP at multiple thresholds compared with echocardiography for detecting LVSD in screened general and high‐risk populations combined.

It was not possible to calculate optimal thresholds for NP screening for LVSD in only high‐risk populations as there were not enough studies of this population that provided data at multiple thresholds. However, the pooled accuracy of NT‐proBNP in high‐risk and general community populations combined (*Figure* *4*) gave an optimal cut‐off of 311 pg/mL with sensitivity of 0.74 (95% CI 0.53–0.88) and specificity 0.85 (95% CI 0.68–0.93). The pooled accuracy data for BNP (*eFigure* *6*) yielded an optimal screening threshold for the detection of LVSD at 49 pg/mL with a sensitivity of 0.68 (95% CI 0.45–0.85) and a specificity of 0.81 (0.67–0.90).

### Sensitivity analysis

Sensitivity analysis demonstrated that overall NP performance was similar when studies that excluded participants with a previous diagnosis of LVSD were compared with studies that did not (*eFigures* *7* *–* *10*). Seven of the included LVSD studies described the screened population as entirely asymptomatic and sensitivity analysis examined whether there were any differences when results of these studies were compared to the other included studies; performance of NP screening was comparable across both groups. Sensitivity analysis was also performed to exclude studies identified as having high risk of bias (*eFigures* *13* *–* *16*), and no major differences in performance were noted. We analysed the available data on women and totals (i.e. men and women combined if reported, and otherwise women only) in a further sensitivity analysis, as we had decided to base our primary analysis on all the available data for men and totals (i.e. men and women combined if reported, and otherwise men only) because not all studies reported data for the combined group of men and women together (*eFigures* *17* and *18*). We felt this analysis was particularly relevant given sex‐specific differences in the manifestation of CV diseases including HF.^56^ There was a drop in the sensitivity of NT‐proBNP from 0.72 (95% CI 0.42–0.90) to 0.68 (95% CI 0.36–0.89) and for BNP from 0.62 (95% 0.32 to 0.85) to 0.59 (95% CI 0.30–0.83), whereas specificity was similar [NT‐proBNP 0.82 (95% CI 0.60–0.93) vs. 0.83 (95% CI 0.63–0.94)] and BNP 0.83 (95% CI 0.61–0.94) vs. 0.82 (95% CI 0.64–0.90). The differences in sensitivity were small, however, and in the context of wide CIs, they may not be clinically meaningful.

One of the included studies, Mason et al.^39^ used only one NT‐proBNP threshold set at 1000 pg/mL (which was higher than most other included studies), and no other results at lower thresholds were included. A sensitivity analysis was therefore performed to examine whether excluding this study affected the pooled accuracy results (*eFigure* *19*); there was no significant change in the pooled sensitivity and specificity for detecting LVSD in screened high‐risk populations with Mason et al. excluded.

## Discussion

We found that in studies recruiting high‐risk community populations, screening with NP testing accurately detected LVSD: NT‐proBNP testing demonstrated a high pooled sensitivity of 0.87 (95% CI 0.73–0.94) and specificity of 0.84 (95% CI 0.55–0.96). In studies of general community populations, the pooled sensitivity of NT‐proBNP to detect LVSD was lower at 0.72 (95% CI 0.42–0.90) with specificity 0.82 (95% CI 0.60–0.93). The pooled accuracy of NT‐proBNP in high‐risk and general community populations combined gave an optimal cut‐off of 311 pg/mL with sensitivity of 0.74 (95% CI 0.53–0.88) and specificity 0.85 (95% CI 0.68–0.93). The pooled accuracy data for BNP yielded an optimal screening threshold for the detection of LVSD at 49 pg/mL with a sensitivity of 0.68 (95% CI 0.45–0.85) and a specificity of 0.81 (0.67–0.90).

### Comparison with previous studies

We compared our results with similar studies exploring the use of NP testing in screening non‐presenting patients for LVSD. Although previous systematic reviews have included NP screening, studies from secondary and community populations have been combined,^20^, ^57^ or community settings were analysed but specifically in nursing homes only.^58^ Ewald et al. calculated a pooled diagnostic odds ratio (DOR) of 9.3 (95% CI 4.7–17.4) for NT‐proBNP screening for severe LVSD.^20^ Based on the pooled estimate of sensitivity and specificity, our DOR for NT‐proBNP to detect LVSD is higher at 42, but there is more heterogeneity within our studies. A recent individual patient data study has also developed and validated a prediction model that identified older patients with HF.^59^ The clinical model improved with the addition of NT‐proBNP and modelling in combination with NP screening may provide the most accurate screening strategy.

Our results for optimal screening thresholds are consistent with previous research in this area. For general and high‐risk populations combined, the optimal threshold for BNP was 49 pg/mL in our study. This aligns closely with the STOP‐HF study that used BNP ≥ 50 pg/mL as a cut‐off for intervention, and it is the results from this study that underpin the current North American guideline recommendations.^13^, ^14^ Moreover, findings from another meta‐analysis of screening studies that combined primary and secondary care studies found that the optimal sensitivity was achieved when BNP was below the cut point of 50 pg/mL.^57^ To put this in context, this level of BNP threshold is similar to 2021 European Society of Cardiology (ESC) guidelines for the diagnosis of chronic HF for patients who present with symptoms in the non‐acute settings in which the upper limit of normal for BNP is 35 pg/mL (125 pg/mL for NT‐proBNP) although these do not refer to screened community populations.^4^ However, the optimal threshold for NT‐proBNP in our analysis of 311 pg/mL was notably higher than the current recommended ESC guideline cut‐off of 125 pg/mL. NT‐proBNP is increasingly used in favour of BNP in some countries, so agreement on an optimal threshold is needed.^60^ A recent German study found that there was an age‐related incremental increase in NT‐proBNP levels in asymptomatic older adults, with significant sex differences also observed.^61^ The Heart Failure Association of the ESC has published practical guidance on the use and interpretation of NP tests, including in the context of screening.^62^

Overall, there is a paucity of HF diagnostic accuracy studies in screened community‐based populations. More research has been conducted in acute HF with more published diagnostic accuracy data available. A meta‐analysis by Roberts et al., which informed the current National Institute for Health and Care Excellence acute HF guideline, pooled data from 37 acute HF diagnostic accuracy studies, including 15 263 NP test results, to evaluate NP test performance.^63^ They found the NP thresholds defined in the ESC HF guideline 2012 performed well, particularly at the lower threshold (BNP < 100 pg/mL, NT‐proBNP<300 pg/mL) for ruling out acute HF.^63^ A similar increase in the number and quality of community‐based screening studies is needed to further evaluate the role of NP testing to detect or rule out LVSD.^4^

### Implications for policy and practice

There is an urgent need for further prospective studies as well as an individual participant data meta‐analysis to more precisely evaluate diagnostic accuracy and identify optimal screening thresholds in specifically defined sub‐populations within the community, including comparing performance by EF, in populations who are aged under or over 65 years, and this should include comparisons of accuracy in men and women. Biological variables such as BMI and renal function also need to be better understood in a screening context.

Current policy on the use of NP screening to detect LVSD varies globally. European guidelines do not recommend NP screening, whereas North American guidelines advocate screening without specifying where the positivity threshold should be set.^14^ Our results provide evidence to support NP screening in high‐risk populations to detect LVSD and contribute to the evidence base on screening thresholds. The performance of NP screening to detect LVSD is comparative in accuracy to some cancer screening approaches such as faecal immunochemical testing (FIT) for haemoglobin to detect colorectal cancer. The FIT test is now integrated into the UK national bowel cancer screening programme.^64^

Screening in healthcare remains a contentious issue. The costs and benefits of any screening programme would need to be considered on a national level. In many European countries, echocardiography services are already under strain and the potential for overdiagnosis when imaging large numbers of people is considerable. Future research should investigate whether false positive rates could be reduced with refinement of target populations, potentially through improved risk prediction, as well as to explore the acceptability of screening from a patient perspective.^65^ The burden on the patient and healthcare system needs to be outweighed by improvements in mortality and quality of life and reduced costs of caring for patients with advanced HF, which may result from earlier diagnosis and treatment. Future research needs to examine both the impact of NP screening for LVSD on patient outcomes and the resources required for management of screen‐detected patients, including up‐titration of medications.^66^

### Strengths and limitations

To our knowledge, this is the first systematic review to focus on the diagnostic accuracy of NP testing in screened community populations alone for the detection of LVSD to compare performance in both high‐risk and general populations and to analyse NP thresholds used in screening studies. Decisions regarding inclusion criteria were based on a pragmatic approach underpined by clinical experience of community diagnostics. To increase applicability to a broad range of clinical contexts, we expanded on the definition of high‐risk populations that were used in STOP‐HF.^13^ This review has been produced in accordance with the PRISMA guidelines and the Cochrane Handbook for Systematic Reviews of Diagnostic Test Accuracy.^23^ There was significant heterogeneity among the included studies due to variation in the included clinical populations, definition of LVSD used by investigators, and disparities in reporting of diagnostic accuracy parameters at different NP thresholds. Study populations differed in the ages of included participants, presence of symptoms, and the prevalence of LVSD. We were unable to undertake planned subgroup analysis by age due to the wide variation in age ranges recruited across studies (see *Table* *1*, *eTable* *1*, and *eTable* *2* for age definitions) and the unavailability of individual patient data. Attempts were made to mitigate for this by analysing data separately for high‐risk and general populations, an approach not taken by most previous systematic reviews that have focussed on NP screening, and examined the population as a whole.^20^, ^21^ We included all studies from screened community populations but only some studies (*n* = 7, 29%) described participants as entirely asymptomatic. Many patients with HF describe being unaware of symptoms initially, particularly when these are very mild, and therefore do not present to a healthcare professional.^5^ The presence or absence of symptoms is therefore variable, and the included studies are aligned with the real‐world experience of community screening where there might be symptomatic patients still presenting for a screening test, particularly if symptoms are mild.

The definitions of LVSD are listed under target condition in *Table* *1*, *eTable* *1*, and *eTable* *2*. As the overall aim of community screening is to identify previously undetected LVSD, we chose to include both narrative and quantitative descriptions of reduced left ventricular EF although this may have contributed to the clinical heterogeneity. We planned to analyse test performance by EF (e.g. <40%, <50%), but there were too few studies to enable this subgroup analysis to be performed.

The different NP thresholds reported by included studies are also listed in *Table* *1*, *eTable* *1*, and *eTable* *2*, all forest plots were also ordered by threshold so that different studies that reported performance at similar thresholds were grouped together. We have attempted to provide an estimation of where optimal screening thresholds might lie. The statistical model that was used to pool sensitivity and specificity to provide a summary estimate is different to the model that is required to identify the optimal threshold.^30^ Fitting this model requires studies to report results at multiple thresholds, and there were not enough such studies in the subgroup of studies in high‐risk populations to allow this. The inability to recommend an optimal screening threshold in high‐risk populations is a major study limitation. To provide an indication of where the appropriate positivity threshold might lie, we estimated an optimal threshold from the pooled high‐risk and general population studies combined. We acknowledge that this also limits the current clinical applicability of the findings although identifying this evidence gap is an important finding and provides a focus for future research that can build on this more general exploration of the accuracy of NP screening for LVSD.

## Conclusions

In high‐risk community populations, it is likely that NP screening may accurately detect LVSD. Given the huge public health burden of missed HF diagnoses in the community, this finding presents a potentially important opportunity for diagnosis and early intervention. Our study highlights an urgent need for further prospective studies, as well as an individual participant data meta‐analysis, to evaluate diagnostic accuracy more precisely, to identify optimal screening thresholds in specifically defined sub‐populations within the community, and to further examine the impact of NP screening on both general and high‐risk populations.

## Conflict of interest

All authors have completed the ICMJE uniform disclosure form at www.icmje.org/coi_disclosure.pdf and declare: CT reports speaker fees from Vifor and Novartis and non‐financial support from Roche outside the submitted work. FDRH reports personal fees and other from Novartis and Boehringer Ingelheim and grants from Pfizer outside the submitted work. CG and the other authors have nothing to disclose.

## Funding

This work was supported by Wellcome Trust Doctoral Fellowships [CG 203921, NJ 203921]. AR is funded by the NIHR Oxford Biomedical Research Centre, Oxford University Hospitals NHS Foundation Trust. KST receives funding from the NIHR Programme Grants for Applied Research. TF receives funding from the National Institute for Health Research Community Healthcare MedTech and In Vitro Diagnostics Co‐operative (MIC) at Oxford Health NHS Foundation Trust. RH acknowledges part‐funding from the NIHR School for Primary Care Research, the NIHR Collaboration for Leadership in Health Research and Care (CLARHC) Oxford, the NIHR Oxford BRC, and the NIHR Oxford MIC. CT is an NIHR Academic Clinical Lecturer. This work is part of the Long Term Condition theme of the NIHR Oxford MIC, which is co‐lead by RH and CT. The views expressed are those of the authors and not necessarily those of the NHS, the NIHR, or the Department of Health and Social Care.

## Supporting information


**Appendix S1.** Search Strategy.
