# Supplementary material for: Diagnostic accuracy of natriuretic peptide screening for left ventricular systolic dysfunction in the community: systematic review and meta‐analysis
Source: ESC Heart Fail. 2023 Feb 13;10(3):1643–55. doi: 10.1002/ehf2.14314 (PMC10192243; doi:10.1002/ehf2.14314)
Supplement: Supplementary file 1 — Appendix S1. Search Strategy. [file EHF2-10-1643-s001.pdf]

Appendix 1: Search Strategy 3

eTable 1: Characteristics of included studies 5

eFigure 1: Methodological quality of included studies: QUADAS-2 risk of bias and applicability shown as a percentage of included studies 9

eFigure 2: Methodological quality of included studies: QUADAS-2 risk of bias summary 10

eTable 2: Full 2 x 2 Data Table Included Studies 11

eFigure 3: Paired sensitivity and specificity plot for NP compared to echocardiography and cardiac MRI for the detection of LVSD in general screened populations

a) NT-proBNP 22

b) BNP 23

eFigure 4: SROC curve of NT-proBNP at multiple thresholds compared with echocardiography for detecting LVSD in screened general risk populations 24

eFigure 5: SROC curve of BNP at multiple thresholds compared with echocardiography and cardiac MRI for detecting LVSD in general risk populations 25

eFigure 6: SROC curve of BNP at multiple thresholds compared with echocardiography for detecting LVSD in screened general and high-risk populations combined 26

eFigure 7: Sensitivity Analysis. Paired sensitivity and specificity plot for NT-proBNP compared to echocardiography for diagnosis of LVSD in high risk screened populations with only studies that excluded patients with prior diagnosis LVSD 27

eFigure 8: Sensitivity Analysis. Paired sensitivity and specificity plot for NT-proBNP compared to echocardiography for diagnosis of LVSD in high risk screened populations with remaining studies that included screened population and did not exclude prior diagnosis LVSD specifically 27

eFigure 9: Sensitivity Analysis. Paired sensitivity and specificity plot for BNP compared to echocardiography for diagnosis of LVSD in high risk screened populations with only studies that excluded patients with prior diagnosis LVSD 27

eFigure 10: Sensitivity Analysis. Paired sensitivity and specificity plot for BNP compared to echocardiography for diagnosis of LVSD in high risk screened populations with remaining studies that included screened population and did not exclude prior diagnosis LVSD specifically 28

eFigure 11: Sensitivity Analysis: Paired sensitivity and specificity plot for NT-proBNP compared to echocardiography for diagnosis of LVSD in studies that described screened population as asymptomatic 28

eFigure 12: Sensitivity Analysis: Paired sensitivity and specificity plot for BNP compared to echocardiography for diagnosis of LVSD in studies that described screened population as asymptomatic 28

eFigure 13: Sensitivity Analysis. Paired sensitivity and specificity plot for NT-proBNP compared with echocardiography for detecting LVSD in screened high-risk populations with studies at high risk of bias (Betti 2009) removed 29

eFigure 14: Sensitivity Analysis. Paired sensitivity and specificity plot for BNP compared with echocardiography for detecting LVSD in screened high-risk populations with studies at high risk of bias (McDonagh 1998, Lobos Bejarano 2012) removed 29

eFigure 15: Sensitivity Analysis. Paired sensitivity and specificity plot for NT-proBNP compared to echocardiography for the detection of LVSD in general screened populations with studies at high risk of bias (Costello-Boerrigter 2006, De Lemos 2008, Groening 2004) removed 30

eFigure 16: Sensitivity Analysis. Paired sensitivity and specificity plot for BNP compared to echocardiography for the detection of LVSD in general screened populations with studies at high risk of bias (Costello-Boerrigter 2006, De Lemos 2008, Luchner 2000, Vasan 2002) removed 31

eFigure 17: Sensitivity Analysis. SROC curve of NT-proBNP at multiple thresholds compared with echocardiography and cardiac MRI for detecting LVSD in general risk populations based on results for women and totals 32

eFigure 18: Sensitivity Analysis. SROC curve of BNP at multiple thresholds compared with echocardiography and cardiac MRI for detecting LVSD in general risk populations based on results for women and totals 33

eFigure 19: Sensitivity Analysis.

a) SROC curves of NT-proBNP compared with echocardiography for detecting LVSD in screened high-risk populations with Mason 2013 removed 34

b) SROC curves of BNP compared with echocardiography for detecting LVSD in screened high-risk populations with Mason 2013 34

Appendix 1: Search Strategy

# ▲	Searches
1	natriuretic peptide, brain/
2	bnp.mp.
3	nt-probnp.mp.
4	brain-type natriuretic peptide.mp.
5	bnp1-32.mp.
6	bnp-32.mp.
7	bnp77-108.mp.
8	probnp.mp.
9	nt-probnp1-76.mp.
10	natriuretic factor-32.mp.
11	natriuretic peptide type-b.mp.
12	type-b natriuretic peptide.mp.
13	ventricular natriuretic peptide.mp.
14	1 or 2 or 3 or 4 or 5 or 6 or 7 or 8 or 9 or 10 or 11 or 12 or 13
15	(heart failure or dyspnea or shortness of breath or cardiac failure or systolic failure or systolic dysfunction or diastolic failure or diastolic dysfunction or alvsd or lvsd or congestive failure or high-output failure or high output failure or low output failure or low-output failure or right-sided failure or right sided failure or left-sided failure or left sided failure or ventricular failure or auricular failure or atrial failure or myocardial failure or cardiac decompensation or heart decompensation or cardiac insufficiency or myocardial insufficiency or CHF or cardiac edema or paroxysmal dyspnea).mp.
16	14 and 15
17	exp animals/ not humans.sh.
18	16 not 17
19	Mass Screening/
20	screen*.ti,ab.

21	detect*.ti,ab.
22	(asymptomatic or preclinical* or pre-clinical*).ti,ab.
23	((community* or population*) adj2 based*).ti,ab.
24	19 or 20 or 21 or 22 or 23
25	18 and 24

eTable 1: Characteristics of included studies

Study Author (Year)	Study Design	Inclusion Criteria	Exclusion Criteria	Follow-up period
Abhayaratna (2006)	Cross-sectional	Canberra Heart study<br>Invitation from electoral roll (random)<br>Age 60 - 85<br>Population based sample	Institutionalised subjects and those who died/moved from Australian Capital Territory excluded from the study	February 2002 - June 2003
Betti (2009)	Cross-sectional	At least one of:<br>type 2 diabetes, on medication for at least the last 6 months<br>systemic hypertension (systolic ≥ 140 or diastolic ≥ 90)<br>on 2 medications for at least the last 6 months	Hx heart failure<br>Hx acute coronary syndrome<br>Hx MI<br>Known moderate-severe valve disease<br>known congenital heart disease<br>primary or secondary myocardial disease<br>previous severe ventricular arrhythmias<br>chronic AF or flutter<br>severe obstructive lung disease<br>previous PE<br>chronic renal failure (creatinine ≥ 2 mg/dL)	Not stated
Cosin Aguilar (2003)	Cross-sectional	Valencian community general population<br>45-74 years old	None listed	Not stated
Costello-Boerrigter (2006)	Cross-sectional	Age ≥ 45<br>Living in Olmsted County, Minnesota	Not stated, describes clearly defnition of normal subjects	Not stated
De Lemos (2009)	Cross-sectional	Dallas Heart Study<br>Aged 30-65<br>Dallas County Residents<br>Deliberate oversampling to include afro-carribean participants	Self reported hx of CHF<br>Prior MI<br>Valvular abnormalities<br>Serum Cr> 2.0mg/dL	July 2000 - September2002

Study Author (Year)	Study Design	Inclusion Criteria	Exclusion Criteria	Follow-up period
Galasko (2005)	Cross-sectional	Random sample from community, aged ≥45 from 7 practices, including 1392 from general population and 928 high-risk patients	None stated	January 2000 – December 2001
Gavazzi (2014)	Cross-sectional	Ischaemic heart disease<br>Ceebrovascular disease<br>Peripheral vascular disease<br>Diabetes mellitus<br>Hypertension	<55 or >80 years old<br>Congenital heart disease<br>Co-morbidities limiting life expectancy<br>Unable to go to doctor’s office	Not stated
Goetze (2006)	Cross-sectional	Patients were from 4th Copenhagen City Heart Study,<br>a longitudinal cohort study of CVD and risk factors<br>20 years or older (equal numbers of patients in each 5 year stratum)<br>Random sample living within a defined area of Copenhagen invited<br>Repeated over subsequent years	None listed	2001-2003
Goode (2007)	Cross-sectional	High-risk patients: at least one of ischaemic heart disease (IHD),<br>previous MI, AF, diabetes for at least 10 years, hypertension<br>for at least 10 years, taking loop diuretic		Not stated
Groenning (2004)	Cross-sectional	Age 50 - 90 years<br>4 Copenhagen GPs in same urban area	Inability to cooperate eg mental reasons such as dementia<br>Residency in nursing homes	1997-1999
Hebert (2010)	Cross-sectional	Age 40 - 75<br>Diabetes mellitus<br>Randomly selected from computerised practice registry	Previous diagnosis of CHF<br>End stage renal disease (CrCl < 60 ml/min)<br>Echocardiogram in last year or referral for echo	January 2004 - February 2005
Hedberg (2004)	Cross-sectional	75 years old<br>Living in Va¨sterås, Sweden<br>General population	None specified	Not stated
Hobbs (2004)	Cross-sectional	16 randomly selected primary care practice populations,<br>stratified for age and socioeconomic status<br>Four cohorts identified from within each practice:<br>Random sample from those aged >45 years (general popn)<br>Patients with clinical (unvalidated) diagnostic label of HF<br>Patients prescribed diuretics<br>Patients at high-risk of HF (previous MI, angina, HTN or DM)	None specified	Not stated

Study Author (Year)	Study Design	Inclusion Criteria	Exclusion Criteria	Follow-up period
Lobos Bejarano (2012)	Cross-sectional	Multi-centre cross-sectional analysis of consecutive patients across 7 centres in Madrid at high-risk of HF. Inclusion - age≥50, hypertension and one other risk factor including LVH, DM and /or history of IHD. Alternatively included if age≥70 and one of the risk factors	Terminal illness, learning difficulties, severe psychiatric illness, drug misuse	February 2008 - June 2008
Luchner (2000)	Cross-sectional	Subjects had participated in the MONICA study, originating from sex-age-stratified random sample of all German residents of Augsburg study, of this 1010 invited, 672 participated	None specified in this paper	Not stated
Luers (2010)	Cross-sectional	At least one CVD risk factor documented by GP, (HTN, diabetes, FH of CAD), or CAD themselves From 58 practices in and around Goettingen, Germany	None stated	Jan 2003 - June 2004
Lukowicz (2005)	Cross-sectional	Subjects had participated in the MONICA study, originating from sex-age-stratified random sample of all German residents of Augsburg study area, different age ranges/data to Luchner although similarities equal distribution of genders, all age classes, all social groups and residents of urban and rural areas.		1995-1996
Mason (2013)	Cross-sectional	Nested diagnostic accuracy study. Cohort were participants in a prevalence study and RCT. Participants were residents from 33 nursing homes in North-East England aged ≥ 65 years, without terminal disease. No exclusions made on the basis of cognitive capacity, comborbidites or immobility.	Terminal disease	April 2009 – June 2010
McDonagh (1998)	Cross-sectional	Randomly selected 2000 people aged 25-74 from North Glasgow who had participated in the Third Glasgow MONICA survey in 1992.	No exclusions stated	Not stated
Mureddu (2013)	Cross-sectional	Residents in the Lazio region (central Italy) from 4 cities identified from Regional Health Registry and in Rome from population lists of patients living in the 21 neighbourhoods surrounding centres where patients underwent study investigations. Aged 65-84 Part of the PREDICTOR study	None specified	July 2007 - January 2010

Study Author (Year)	Study Design	Inclusion Criteria	Exclusion Criteria	Follow-up period
Murtagh (2012)	Cross-sectional	STOP HF cohort, population Age≥ 40 years and at least one risk factor for LVSD. Patients referred by primary care physician, 39 participating practices<br><br>Hypertension - medicated for ≥1 month); (2) hypercholesterolemia, TC > 193 mg/dL (5.0 mmol/L) (174 mg/Dl [4.5 mmol/L] in high-risk patients) and/or low-density lipo-protein cholesterol greater than 116 mg/dL (3.0 mmol/L) (97 mg/dL [2.5 mmol/L] in high-risk patients) or receiving lipid-lowering therapy; (3) obesity, defined as body mass index (calculated as weight in kilograms divided by height in meters squared) greater than 30; (4) vascular disease, including CHD, CVD, PVD (5) diabetes mellitus; (6) arrhythmia requiring therapy; or (7) moderate to severe valvular disease.	Excluded those who refused to provide informed consent, established evidence of LVSD, had evidence or a history of symptomatic HF or diagnosis compromising survival over the study period.	2005 - 2009
Ng (2003)	Cross-sectional	Randomly selected men (45-80 years)<br>and women (55-80 years) from 21 practices<br>stratified by list size and deprivation score in Leicestershire.	Prior hx of confirmed LVSD or HF / screening inappropriate<br><br>(housebound or terminally ill)	Not stated
Smith (2000)	Cross-sectional	Age 70 – 84	None specified	Not stated
Vasan (2002)	Cross-sectional	Framingham Study- community based prospective cohort, who attended<br>routine exam 1995-1998, List from local census used after stratification<br>for family size and precinct of residence, 6507 in sample<br>4494 included in sample, plus a group of volunteers,<br>no selected, so group was 5209<br>This study includes 3532 subjects who attended 6th exam cycle<br>1995-1998 Patients aged 30-59	Unavailable NP results<br>Creatinine≥2.0/mg/dL, history of heart failure, inadequate<br>echo after these exclusions 3177 remain	1995-1998

Characteristics of included studies. Author, year, study design, inclusion and exclusion criteria, follow up period.

eFigure 1: Methodological quality of included studies: QUADAS-2 risk of bias and applicability shown as a percentage of included studies

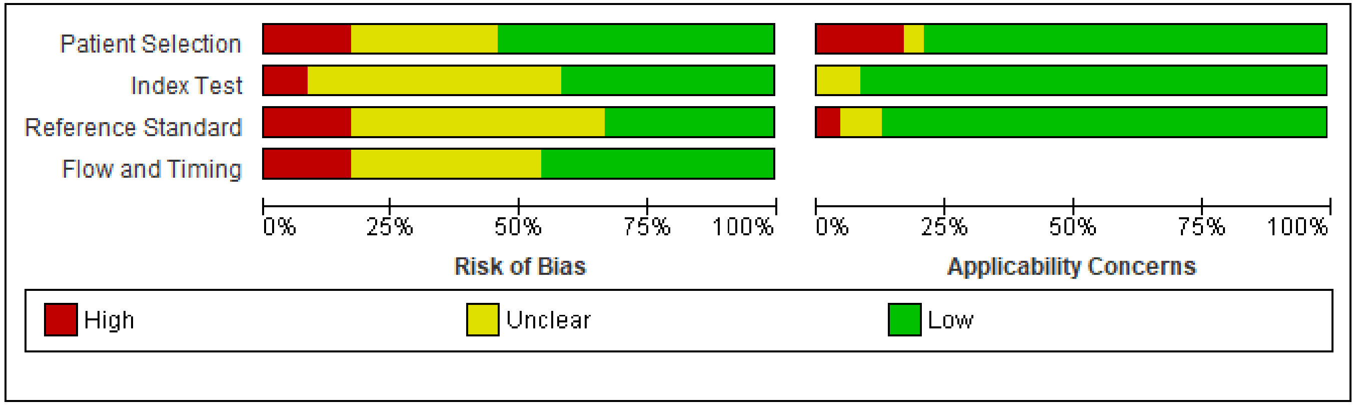

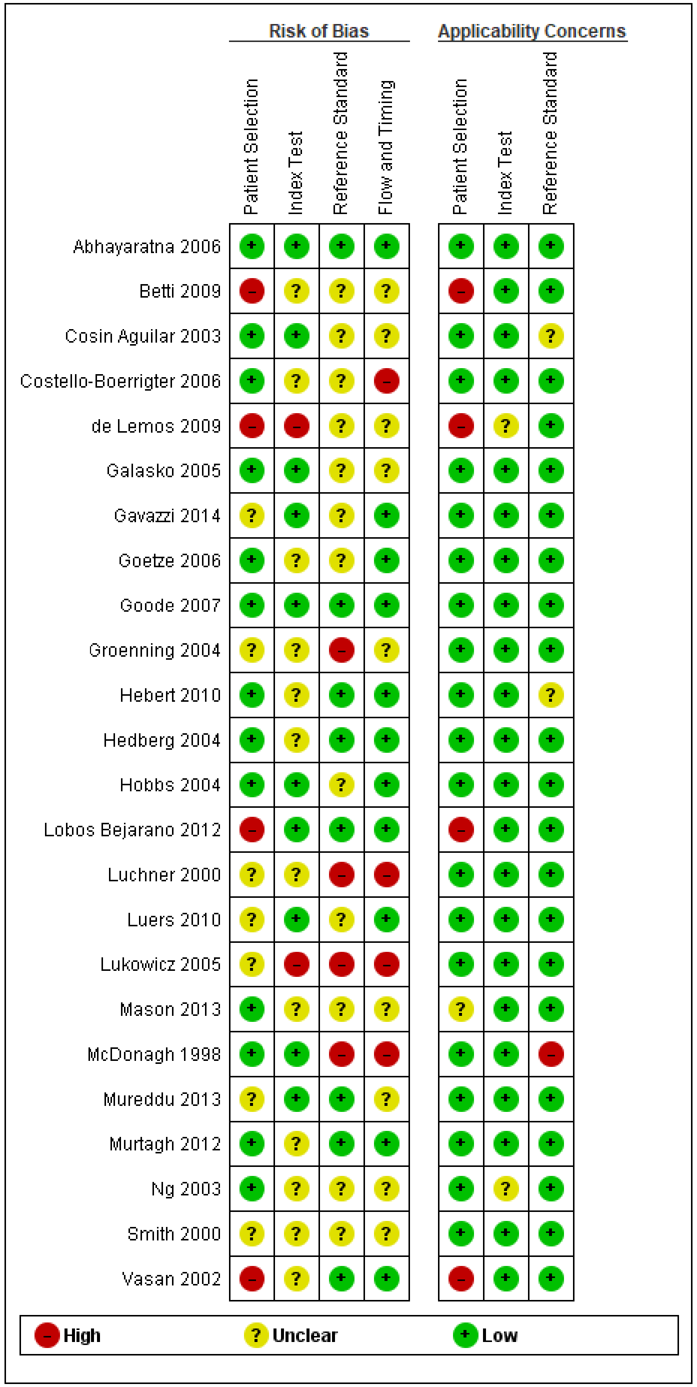

eFigure 2: Methodological quality of included studies: QUADAS-2 risk of bias summary

eTable 2: Full 2 x 2 Data Table Included Studies

Author	Year	Sample size	n	Index Test	Threshold (pg/ml)	Sensitivity	Specificity	Prevalence(%)	Target condition/ Population
Abhayaratna	2006	458	32	NT pro-BNP sandwich ELISA,Elecsys1010 Roche	197	0.84	0.89	7.0	EF ≤ 40 % and advanced DD-NEF* /men 60 - 74
Abhayaratna	2006	155	20	NT pro-BNP sandwich ELISA,Elecsys1010 Roche	452	0.85	0.79	12.9	EF ≤ 40 % and advanced DD-NEF* /men 75 - 86
Abhayaratna	2006	613	21	NT pro-BNP sandwich ELISA,Elecsys1010 Roche	452	0.86	0.91	3.4	LVSD EF ≤ 40 % / men aged 60-86
Abhayaratna	2006	458	54	NT pro-BNP sandwich ELISA,Elecsys1010 Roche	151	0.78	0.88	11.8	EF ≤ 50 % and advanced DD-NEF* /men 60 - 74
Abhayaratna	2006	155	32	NT pro-BNP sandwich ELISA,Elecsys1010 Roche	426	0.81	0.80	20.6	EF ≤ 50 % and advanced DD-NEF* /men 75 - 86
Abhayaratna	2006	613	55	NT pro-BNP sandwich ELISA,Elecsys1010 Roche	206	0.76	0.81	9.0	LVSD EF ≤ 50 % / men aged 60-86
Abhayaratna	2006	470	22	NT pro-BNP sandwich ELISA,Elecsys1010 Roche	270	0.82	0.90	4.7	EF ≤ 40 % and advanced DD-NEF* /women 60 - 74
Abhayaratna	2006	146	23	NT pro-BNP sandwich ELISA,Elecsys1010 Roche	375	0.83	0.83	15.8	EF ≤ 40 % and advanced DD-NEF* /women 75 - 86
Abhayaratna	2006	616	6	NT pro-BNP sandwich ELISA,Elecsys1010 Roche	710	1.00	0.96	1.0	LVSD EF ≤ 40 % / women aged 60-86
Abhayaratna	2006	470	31	NT pro-BNP sandwich ELISA,Elecsys1010 Roche	270	0.68	0.90	6.6	EF ≤ 50 % and advanced DD-NEF* /women 60 - 74
Abhayaratna	2006	146	28	NT pro-BNP sandwich ELISA,Elecsys1010 Roche	375	0.86	0.86	19.2	EF ≤ 50 % and advanced DD-NEF* /women 75 - 86
Abhayaratna	2006	616	20	NT pro-BNP sandwich ELISA,Elecsys1010 Roche	296	0.70	0.84	3.2	LVSD EF ≤ 50 % / women aged 60-86

Author	Year	Sample size	n	Index Test	Threshold (pg/ml)	Sensitivity	Specificity	Prevalence(%)	Target condition/ Population
Betti	2009	204	8	NT-proBNP	125	0.88	0.93	3.9	Pooled systolic and moderate-severeDD / men < 67
Betti	2009	131	13	NT-proBNP	125	1.00	0.77	9.9	Pooled systolic and moderate-severeDD / men ≥ 67
Betti	2009	191	14	NT-proBNP	125	1.00	0.84	7.3	Pooled systolic and moderate-severeDD / women < 67
Betti	2009	159	17	NT-proBNP	125	1.00	0.60	10.7	Pooled systolic and moderate-severeDD / women ≥ 67
Betti	2009	1012	52	NT-proBNP	125	0.98	0.80	5.1	Pooled systolic and moderate-severeDD / all patients
Cosin Aguilar	2003	203	12	NTproBNP (Roche Diagnostics)	319	0.92	0.68	5.9	LVEF ≤50%
Cosin Aguilar	2003	203	12	NTproBNP (Roche Diagnostics)	215	0.92	0.53	5.9	LVEF ≤50%
Cosin Aguilar	2003	203	12	NTproBNP (Roche Diagnostics)	1780	0.25	0.96	5.9	LVEF ≤50%
Costello-Boerrigter	2006	907	29	NT-proBNP sandwich ELISA(Elecsys)	209	0.86	0.87	3.2	EF ≤ 40 % / all men
Costello-Boerriger	2006	557	9	NT-proBNP sandwich ELISA(Elecsys)	109	0.89	0.90	1.6	EF ≤ 40 % / men < 65
Costello-Boerrigter	2006	350	20	NT-proBNP sandwich ELISA(Elecsys)	462	0.86	0.88	5.7	EF ≤ 40 % / men ≥ 65
Costello-Boerrigter	2006	907	85	NT-proBNP sandwich ELISA(Elecsys)	92	0.75	0.75	9.4	EF ≤ 50 % / all men
Costello-Boerrigter	2006	557	35	NT-proBNP sandwich ELISA(Elecsys)	32	0.63	0.63	6.3	EF ≤ 50 % / men < 65

Author	Year	Sample size	n	Index Test	Threshold (pg/ml)	Sensitivity	Specificity	Prevalence(%)	Target condition/ Population
Costello-Boerrigter	2006	350	50	NT-proBNP sandwich ELISA(Elecsys)	211	0.79	0.79	14.3	EF ≤ 50 % / men ≥ 65
Costello-Boerrigter	2006	962	8	NT-proBNP sandwich ELISA(Elecsys)	372	0.83	0.83	0.8	EF ≤ 40 % / all women
Costello-Boerrigter	2006	565	2	NT-proBNP sandwich ELISA(Elecsys)	245	1.00	0.94	0.4	EF ≤ 40 % / women < 65
Costello-Boerrigter	2006	397	6	NT-proBNP sandwich ELISA(Elecsys)	372	0.83	0.83	1.5	EF ≤ 40 % / women ≥ 65
Costello-Boerrigter	2006	962	30	NT-proBNP sandwich ELISA(Elecsys)	246	0.75	0.73	3.1	EF ≤ 50 % / all women
Costello-Boerrigter	2006	565	10	NT-proBNP sandwich ELISA(Elecsys)	105	0.70	0.70	1.8	EF ≤ 50 % / women < 65
Costello-Boerrigter	2006	397	20	NT-proBNP sandwich ELISA(Elecsys)	246	0.75	0.73	5.0	EF ≤ 50 % / women ≥ 65
Costello-Boerrigter	2006	1869	37	NT-proBNP sandwich ELISA(Elecsys)	228	0.87	0.86	2.0	EF ≤ 40 % / all patients
Costello-Boerrigter	2006	1122	11	NT-proBNP sandwich ELISA(Elecsys)	163	0.91	0.91	1.0	EF ≤ 40 % / age < 65
Costello-Boerrigter	2006	747	26	NT-proBNP sandwich ELISA(Elecsys)	452	0.89	0.88	3.5	EF ≤ 40 % / age ≥ 65
Costello-Boerrigter	2006	1869	115	NT-proBNP sandwich ELISA(Elecsys)	129	0.74	0.74	6.2	EF ≤ 50 % / all patients
Costello-Boerrigter	2006	1122	45	NT-proBNP sandwich ELISA(Elecsys)	59	0.62	0.61	4.0	EF ≤ 50 % / age < 65
Costello-Boerrigter	2006	747	70	NT-proBNP sandwich ELISA(Elecsys)	233	0.76	0.76	9.4	EF ≤ 50 % / age ≥ 65
Costello-Boerrigter	2006	907	29	BNP immunoassay (Biosite)	55	0.83	0.82	3.2	EF ≤ 40 % / all men

Author	Year	Sample size	n	Index Test	Threshold (pg/ml)	Sensitivity	Specificity	Prevalence (%)	Target condition/ Population
Costello-Boerrigter	2006	557	9	BNP immunoassay (Biosite)	24	0.78	0.78	1.6	EF ≤ 40 % / men < 65
Costello-Boerrigter	2006	350	20	BNP immunoassay (Biosite)	79	0.75	0.76	5.7	EF ≤ 40 % / men ≥ 65
Costello-Boerrigter	2006	907	85	BNP immunoassay (Biosite)	28	0.71	0.70	9.4	EF ≤ 50 % / all men
Costello-Boerrigter	2006	557	35	BNP immunoassay (Biosite)	12	0.57	0.57	6.3	EF ≤ 50 % / men < 65
Costello-Boerrigter	2006	350	50	BNP immunoassay (Biosite)	61	0.72	0.72	14.3	EF ≤ 50 % / men ≥ 65
Costello-Boerrigter	2006	962	8	BNP immunoassay (Biosite)	102	0.88	0.88	0.8	EF ≤ 40 % / all women
Costello-Boerrigter	2006	565	2	BNP immunoassay (Biosite)	108	1.00	0.96	0.4	EF ≤ 40 % / women < 65
Costello-Boerrigter	2006	397	6	BNP immunoassay (Biosite)	124	0.83	0.81	1.5	EF ≤ 40 % / women ≥ 65
Costello-Boerrigter	2006	962	30	BNP immunoassay (Biosite)	57	0.73	0.73	3.1	EF ≤ 50 % / all women
Costello-Boerrigter	2006	565	10	BNP immunoassay (Biosite)	30	0.60	0.60	1.8	EF ≤ 50 % / women < 65
Costello-Boerrigter	2006	397	20	BNP immunoassay (Biosite)	79	0.70	0.70	5.0	EF ≤ 50 % / women ≥ 65
Costello-Boerrigter	2006	1869	37	BNP immunoassay (Biosite)	66	0.81	0.81	2.0	EF ≤ 40 % / all patients
Costello-Boerrigter	2006	1122	11	BNP immunoassay (Biosite)	42	0.82	0.82	1.0	EF ≤ 40 % / age < 65
Costello-Boerrigter	2006	747	26	BNP immunoassay (Biosite)	97	0.77	0.77	3.5	EF ≤ 40 % / age ≥ 65

Author	Year	Sample size	n	Index Test	Threshold (pg/ml)	Sensitivity	Specificity	Prevalence (%)	Target condition/ Population
Costello-Boerrigter	2006	1869	115	BNP immunoassay (Biosite)	40	0.68	0.69	6.2	EF ≤ 50 % / all patients
Costello-Boerrigter	2006	1122	45	BNP immunoassay (Biosite)	19	0.56	0.56	4.0	EF ≤ 50 % / age < 65
Costello-Boerrigter	2006	747	70	BNP immunoassay (Biosite)	73	0.71	0.71	9.4	EF ≤ 50 % / age ≥ 65
De Lemos	2009	1069	nr	NTproBNP (Elecsys, Roche,Indianapolis, USA)	102.5	0.27	0.96	9.0	LVSD EF<55 or LVH / Men
De Lemos	2009	1069	nr	NTproBNP (Elecsys, Roche,Indianapolis, USA)	28.9	0.57	0.71	9.5	LVSD EF<55 or LVH / Men
De Lemos	2009	1069	nr	NTproBNP (Elecsys, Roche,Indianapolis, USA)	52.3	0.42	0.88	8.8	LVSD EF<55 or LVH / Men
De Lemos	2009	1069	nr	NTproBNP (Elecsys, Roche,Indianapolis, USA)	75.2	0.35	0.93	8.4	LVSD EF<55 or LVH / Men
De Lemos	2009	1069	nr	NTproBNP (Elecsys, Roche,Indianapolis, USA)	155.3	0.16	0.98	9.2	LVSD EF<55 or LVH / Men
De Lemos	2009	1360	nr	NTproBNP (Elecsys, Roche,Indianapolis, USA)	66	0.52	0.72	4.4	LVSD EF<55 or LVH / Women
De Lemos	2009	1360	nr	NTproBNP (Elecsys, Roche,Indianapolis, USA)	103.2	0.43	0.87	4.5	LVSD EF<55 or LVH / Women
De Lemos	2009	1360	nr	NTproBNP (Elecsys, Roche,Indianapolis, USA)	139.3	0.29	0.93	5.2	LVSD EF<55 or LVH / Women
De Lemos	2009	1360	nr	NTproBNP (Elecsys, Roche,Indianapolis, USA)	179.6	0.21	0.96	4.8	LVSD EF<55 or LVH / Women
De Lemos	2009	1360	nr	NTproBNP (Elecsys, Roche,Indianapolis, USA)	256.1	0.16	0.99	4.7	LVSD EF<55 or LVH / Women
De Lemos	2009	1069	nr	BNP (Biosite Inc, San Diego, CA)	7.1	0.44	0.73	8.9	LVSD EF<55 or LVH / Men

Author	Year	Sample size	n	Index Test	Threshold (pg/ml)	Sensitivity	Specificity	Prevalence (%)	Target condition/ Population
De Lemos	2009	1069	nr	BNP (Biosite Inc, San Diego, CA)	18	0.22	0.89	9.0	LVSD EF<55 or LVH / Men
De Lemos	2009	1069	nr	BNP (Biosite Inc, San Diego, CA)	25.2	0.16	0.94	8.9	LVSD EF<55 or LVH / Men
De Lemos	2009	1069	nr	BNP (Biosite Inc, San Diego, CA)	34.3	0.12	0.97	8.7	LVSD EF<55 or LVH / Men
De Lemos	2009	1069	nr	BNP (Biosite Inc, San Diego, CA)	49.5	0.08	0.99	8.6	LVSD EF<55 or LVH/ Men
De Lemos	2009	1360	nr	BNP (Biosite Inc, San Diego, CA)	13.8	0.49	0.73	5.6	LVSD EF<55 or LVH / Women
De Lemos	2009	1360	nr	BNP (Biosite Inc, San Diego, CA)	26.5	0.31	0.88	5.0	LVSD EF<55 or LVH / Women
De Lemos	2009	1360	nr	BNP (Biosite Inc, San Diego, CA)	39.5	0.19	0.94	4.6	LVSD EF<55 or LVH / Women
De Lemos	2009	1360	nr	BNP (Biosite Inc, San Diego, CA)	53.2	0.15	0.97	4.5	LVSD EF<55 or LVH / Women
De Lemos	2009	1360	nr	BNP (Biosite Inc, San Diego, CA)	65.5	0.12	0.99	4.5	LVSD EF<55 or LVH / Women
Galasko	2005	1205	41	NTproBNP (Elecsys 2010 Roach Diagnostics, Lewes, UK)	age/sex upper ref values	0.88	0.79	3.4	LVEF<40% / all participants
Galasko	2005	734	15	NTproBNP (Elecsys 2010 Roach Diagnostics, Lewes, UK)	age/sex upper ref values	1.00	0.88	2.0	LVEF<40% / general population
Galasko	2005	761	41	NTproBNP (Elecsys 2010 Roach Diagnostics, Lewes, UK)	age/sex upper ref values	0.88	0.69	5.4	LVEF<40% / high risk subjects
Galasko	2005	1205	105	NTproBNP (Elecsys 2010 Roach Diagnostics, Lewes, UK)	age/sex upper ref values	0.63	0.81	8.7	LVEF<50% / all participants

Author	Year	Sample size	n	Index Test	Threshold (pg/ml)	Sensitivity	Specificity	Prevalence (%)	Target condition/ Population
Galasko	2005	734	39	NTproBNP (Elecsys 2010 Roach Diagnostics, Lewes, UK)	age/sex upper ref values	0.66	0.89	5.3	LVEF<50% / general population
Galasko	2005	761	98	NTproBNP (Elecsys 2010 Roach Diagnostics, Lewes, UK)	age/sex upper ref values	0.66	0.71	12.9	LVEF<50% / high risk
Gavazzi	2014	619	9	NTproBNP (Cardiac Reader, Roche Diagnostics, Mannheim, Germania)	age/sex 95th percentile	0.72	1.00	1.5	LVEF < 40% /high CV risk (IHD / post MI / CVD / arterial hypertension)
Gavazzi	2014	619	20	NTproBNP (Cardiac Reader, Roche Diagnostics, Mannheim, Germania)	age/sex 95th percentile	0.73	0.90	3.2	LVEF < 45% / high CV risk (IHD/post MI/ CVD/ htn)
Gavazzi	2014	619	33	NTproBNP (Cardiac Reader, Roche Diagnostics, Mannheim, Germania)	age/sex 95th percentile	0.70	0.73	5.3	LVEF < 50% / high CV risk (IHD/pos t MI/ CVD/ htn)
Goetze	2006	1502	9	NTproBNP	144	1.00	0.56	0.6	LVEF < 40 % / men
Goetze	2006	1502	9	NTproBNP	626	0.33	0.95	0.6	LVEF < 40 % / men
Goetze	2006	1995	5	NTproBNP	254	1.00	0.66	0.3	LVEF < 40 % / women
Goetze	2006	1995	5	NTproBNP	651	0.60	0.95	0.3	LVEF < 40 % / women
Goode	2007	427	32	NT-proBNP	150	0.84	0.45	7.5	LVEF < 40 % / Patients at high risk LVSD
Goode	2007	427	32	NT-proBNP	age/sex 97.5th percentile	0.84	0.53	7.5	LVEF < 40 % / Patients at high risk LVSD
Goode	2007	427	32	Logistic regression model (NT-proBNP, QRS duration, previous MI and presence of symptoms)	sens 100	1.00	0.54	7.5	LVEF < 40 % / Patients at high risk LVSD
Goode	2007	427	32	Logistic regression model (NT-proBNP, QRS duration, previous MI and presence of symptoms)	sens 84.4	0.84	0.82	7.5	LVEF < 40 % / Patients at high risk LVSD

Author	Year	Sample size	n	Index Test	Threshold (pg/ml)	Sensitivity	Specificity	Prevalence (%)	Target condition/ Population
Groenning	2004	672	21	NT-proBNP sandwich ELISA	850	0.76	0.85	3.1	LVSD LVEF ≤ 35% /all patients
Groenning	2004	672	38	NT-proBNP sandwich ELISA	414	0.76	0.67	5.7	LVSD LVEF ≤ 40% /all patients
Groenning	2004	672	58	NT-proBNP sandwich ELISA	366	0.74	0.64	8.6	LVSD LVEF ≤ 45% / all patients
Groenning	2004	672	77	NT-proBNP sandwich ELISA	351	0.70	0.63	11.5	LVSD LVEF ≤ 50% /all patients
Hebert	2010	145	16	Triage BNP (BioSite, San Diego, USA)	60	0.67	0.85	11.0	LVEF <= 54 % / all patients
Hedberg	2004	407	28	BNP	28	0.93	0.55	6.9	LVSD based on wall motion index corresponding to LVEF < 40 % / all patients
Hedberg	2004	407	28	BNP	73	0.79	0.89	6.9	LVSD based on wall motion index corresponding to LVEF < 40 % / all patients
Hobbs	2004	307	5	NTproBNP (Roche)	338	0.80	0.73	1.6	LVSD < 40% / general population over45 years
Hobbs	2004	87	7	NTproBNP (Roche)	338	0.86	0.40	8.0	LVSD< 40% / patients on diuretics
Hobbs	2004	87	7	BNP	103.8	0.86	0.65	8.0	LVSD< 40% / patients on diuretics
Hobbs	2004	307	5	BNP	103.8	0.80	0.88	1.6	LVSD< 40%/ general population over 45 year
Hobbs	2004	133	10	BNP	103.8	0.50	0.67	7.5	LVSD< 40% / patients at high risk for heart failure
Hobbs	2004	133	10	NTproBNP (Roche)	338	1.00	0.46	7.5	LVSD< 40% / patients at high risk for heart failure
Lobos Bejarano	2012	192	nr	BNP (Biosite Triage)	71	0.75	0.70	9.0	LVSD EF<50%, high risk of presenting HF, Stage A and B of AHA ACC / hypertension, diabetes, age > 70 years, LVH

Author	Year	Sample size	n	Index Test	Threshold (pg/ml)	Sensitivity	Specificity	Prevalence (%)	Target condition/ Population
Luchner	2000	610	39	BNP	34	0.28	0.86	6.4	LVD (defined as fractional shortening <28%) / general population
Luers	2010	542	23	NT-proBNP	380	0.65	0.88	4.2	LVSD (LVEF < 50 %) / all patients (inc all had one CVD RF)
Luers	2010	542	23	Risk score incorporating NT-proBNP	nr	0.91	0.71	4.2	LVSD (defined as LVEF < 50 %) / all patients (inc all had one CVD RF)
Luers	2010	542	nr	Risk score incorporating NT-proBNP	nr	0.90	0.72	6.8	LVSD or severe DD / all patients (incl all had one CVD RF)
Luers	2010	542	nr	NT-proBNP	380	0.63	0.89	6.9	LVSD or severe DD / all patients(included patients all had one CVD RF)
Luers	2010	542	nr	BNP	58	0.74	0.75	8.2	LVSD or DD / all patients (incl all had one CVD RF)
Luers	2010	542	23	BNP	105	0.61	0.90	4.2	LVSD (LVEF < 50 %) / all patients (inc all had one CVD RF)
Lukowicz	2005	1160.714	65	BNP (Shionogi, Japan)	10.2	0.80	0.61	5.6	LVH
Lukowicz	2005	1123	5	BNP (Shionogi, Japan)	27	0.80	0.90	0.4	LVSD (EF< 40%) /general population
Mason	2013	392	34	BNP	145	0.76	0.75	8.7	LVSD EF<50%
Mason	2013	393	34	NT-proBNP	1000	0.73	0,76	8.7	LVSD EF<50%
McDonagh	1998	1252	nr	BNP (RIS kit for human, RIK 9086, Peninsula labs, USA)	17.9	0.76	0.87	3.2	LVSD<30% / General population aged 25-74
McDonagh	1998	1252	nr	BNP (RIS kit for human, RIK 9086, Peninsula labs, USA)	17.9	0.84	0.76	11.0	LVSD<30% / With IHD, aged 25-74
Mureddu	2013	1452	945	NTproBNP (Elecys Roach 2010, Roche Diagnostics GmbH)	278	0.14	0.93	65.1	Stage B HF / Whole population

Author	Year	Sample size	n	Index Test	Threshold (pg/ml)	Sensitivity	Specificity	Prevalence (%)	Target condition/ Population
Mureddu	2013	1452	22	NTproBNP (Elecys Roach 2010, Roche Diagnostics GmbH)	278	0.64	0.89	1.5	LVSD EF<50 / Whole population
Mureddu	2013	435	337	NTproBNP (Elecys Roach 2010, Roche Diagnostics GmbH)	278	0.23	0.89	77.5	Stage B HF/ High risk population
Mureddu	2013	435	6	NTproBNP (Elecys Roach 2010, Roche Diagnostics GmbH)	278	1.00	0.81	1.4	LVSD EF<50 / High risk population
Murtagh	2012	814	33	BNP (Triage Meter POCT assay, Biosite, San Diego, USA)	20	0.88	0.46	4.1	LVSD < 50% / >=1 risk factor
Murtagh	2012	814	11	BNP (Triage Meter POCT assay, Biosite, San Diego, USA)	50	0.70	0.77	1.4	LVSD < 40% />= 1 risk factor
Ng	2003	1331	17	BNP (Peninsular labs, USA)	66	0.97	0.443	1.3	Definite systolic EF ≤ 35%/ general population
Ng	2003	1331	30	BNP (Peninsular labs, USA)	66	1.00	0.30	2.3	Definite and borderline systolic EF ≤ 35% / general population
Ng	2003	1331	17	N-BNP Non-compeitive immunoluminometric assay	318	1.00	0.47	1.3	Definite systolic EF ≤ 35% / general population
Ng	2003	1331	30	N-BNP Non-compeitive immunoluminometric assay	48	0.98	0	2.3	Definite and borderline systolic HF EF ≤ 35% / general population
Smith	2000	155	12	BNP	92.4	0.75	0.8	7.7	Left ventricular systolic dysfunction / all patients
Smith	2000	155	12	BNP	68.5	0.83	0.70	7.7	Left ventricular systolic dysfunction / all patients
Smith	2000	155	12	BNP	64.7	0.92	0.65	7.7	Left ventricular systolic dysfunction / all patients
Vasan	2002	1470	137	BNP (ShionoRIA, Shionogi Inc, Osaka, Japan)	21	0.53	0.84	9.3	Maximising sum of sens and spec / Any LVSD / Men
Vasan	2002	1470	137	BNP (ShionoRIA, Shionogi Inc, Osaka, Japan)	45	0.29	0.95	9.3	95% Spec / Any LVSD / Men

**Author**	**Year**	**Sample size**	**n**	**Index Test**	**Threshold (pg/ml)**	**Sensitivity**	**Specificity**	**Prevalence (%)**	**Target condition/ Population**
Vasan	2002	1470	60	BNP (ShionoRIA, Shionogi Inc, Osaka, Japan)	51	0.33	0.95	4.1	95% Spec/Mod to severe LVSD / Men
Vasan	2002	1470	60	BNP (ShionoRIA, Shionogi Inc, Osaka, Japan)	24	0.65	0.86	4.1	Max sum of sens and spec / Mod to Severe LVSD / Men
Vasan	2002	1470	60	BNP (ShionoRIA, Shionogi Inc, Osaka, Japan)	nr	0.55	0.89	4.1	Age & sex specific reference limits for BNP/Moderate to Severe LVSD/Men
Vasan	2002	1470	137	BNP (ShionoRIA, Shionogi Inc, Osaka, Japan)	nr	0.42	0.90	9.3	Age & sex specific reference limits for BNP/Any LVSD/Men
Vasan	2002	1707	42	BNP (ShionoRIA, Shionogi Inc, Osaka, Japan)	50	0.14	0.95	2.5	95% Spec/Any LVSD / Women
Vasan	2002	1707	10	BNP (ShionoRIA, Shionogi Inc, Osaka, Japan)	50	0.40	0.95	0.6	95% Spec/Moderate to severe LVSD / Women
Vasan	2002	1707	42	BNP (ShionoRIA, Shionogi Inc, Osaka, Japan)	34	0.26	0.89	2.5	Max sum of sens and spec / Any LVSD / Women
Vasan	2002	1707	10	BNP (ShionoRIA, Shionogi Inc, Osaka, Japan)	34	0.80	0.90	0.6	Max sum of sens and spec / Mod to Severe LVSD / Women
Vasan	2002	1707	42	BNP (ShionoRIA, Shionogi Inc, Osaka, Japan)	nr	0.17	0.92	2.5	Age & sex specific reference limits for BNP/Any LVSD/Women
Vasan	2002	1707	10	BNP (ShionoRIA, Shionogi Inc, Osaka, Japan)	nr	0.50	0.93	0.6	Age & sex specific reference limits for BNP/Mod to Severe LVSD/ Women

n: number with outcome, nr not reported *DD-NEF diastolic dysfunction LV EF(EF>50%), DD diastolic dysfunction

eFigure 3: Paired sensitivity and specificity plot for NP compared to echocardiography and cardiac MRI for the detection of LVSD in general screened populations
*Galasko thresholds based on age/sex specific 97.5th percentiles: 100pg/ml males aged 45-59; 164pg/ml females aged 45-59; 172pg/ml males aged >=60 ; 225pg/ml females age>=60.

a) NT-proBNP

Study	TP	FP	FN	TN	Age group	Sex	Threshold	Sensitivity (95% CI)	Specificity (95% CI)
Galasko 2005a	9	99	21	605	>=45	Total	*	0.30 [0.15, 0.49]	0.86 [0.83, 0.88]
Galasko 2005b	15	86	0	633	>=45	Total	*	1.00 [0.78, 1.00]	0.88 [0.85, 0.90]
Galasko 2005c	26	76	13	619	>=45	Total	*	0.67 [0.50, 0.81]	0.89 [0.87, 0.91]
De Lemos 2008f	58	280	44	687	<65	Males	28.0	0.57 [0.47, 0.67]	0.71 [0.68, 0.74]
Ng 2003	29	1301	1	0	60-74	Total	48.0	0.97 [0.83, 1.00]	0.00 [0.00, 0.00]
De Lemos 2008g	40	117	55	858	<65	Males	52.0	0.42 [0.32, 0.53]	0.88 [0.86, 0.90]
De Lemos 2008a	31	364	29	936	<65	Females	66.0	0.52 [0.38, 0.65]	0.72 [0.69, 0.74]
De Lemos 2008h	31	69	58	911	<65	Males	75.0	0.35 [0.25, 0.46]	0.93 [0.91, 0.94]
De Lemos 2008i	26	39	70	934	<65	Males	102.0	0.27 [0.19, 0.37]	0.96 [0.95, 0.97]
De Lemos 2008b	26	169	35	1130	<65	Females	103.0	0.43 [0.30, 0.56]	0.87 [0.85, 0.89]
Costello-Boerrigter 2006d	8	55	1	493	<65	Males	109.0	0.89 [0.52, 1.00]	0.90 [0.87, 0.92]
De Lemos 2008c	20	90	50	1199	<65	Females	139.0	0.29 [0.18, 0.41]	0.93 [0.91, 0.94]
Goetze 2006c	9	657	0	836	>55	Males	144.0	1.00 [0.66, 1.00]	0.56 [0.53, 0.59]
De Lemos 2008j	16	19	83	951	<65	Males	155.0	0.16 [0.10, 0.25]	0.98 [0.97, 0.99]
Costello-Boerrigter 2006g	10	101	1	1010	<65	Total	163.0	0.91 [0.59, 1.00]	0.91 [0.89, 0.93]
De Lemos 2008d	14	52	52	1243	<65	Females	179.0	0.21 [0.12, 0.33]	0.96 [0.95, 0.97]
Abhayaratna 2006c	27	47	5	379	60-74	Males	197.0	0.84 [0.67, 0.95]	0.89 [0.86, 0.92]
Costello-Boerrigter 2006e	25	111	4	767	>=45	Males	209.0	0.86 [0.68, 0.96]	0.87 [0.85, 0.89]
Cosin Aguilar 2003a	11	90	1	101	49-81	Total	215.0	0.92 [0.62, 1.00]	0.53 [0.46, 0.60]
Costello-Boerrigter 2006h	32	256	5	1576	>=50	Total	228.0	0.86 [0.71, 0.95]	0.86 [0.84, 0.88]
Costello-Boerrigter 2006a	2	32	0	531	<65	Females	245.0	1.00 [0.16, 1.00]	0.94 [0.92, 0.96]
Goetze 2006a	5	677	0	1313	>55	Females	254.0	1.00 [0.48, 1.00]	0.66 [0.64, 0.68]
De Lemos 2008e	10	13	53	1283	<65	Females	256.0	0.16 [0.08, 0.27]	0.99 [0.98, 0.99]
Abhayaratna 2006a	18	46	4	402	60-74	Females	270.0	0.82 [0.60, 0.95]	0.90 [0.87, 0.92]
Mureddu 2013	14	157	8	1273	65-84	Total	278.0	0.64 [0.41, 0.83]	0.89 [0.87, 0.91]
Cosin Aguilar 2003b	11	61	1	130	49-81	Total	319.0	0.92 [0.62, 1.00]	0.68 [0.61, 0.75]
Hobbs 2004	4	82	1	220	>=45	Total	338.0	0.80 [0.28, 0.99]	0.73 [0.67, 0.78]
Costello-Boerrigter 2006b	7	159	1	795	>=45	Females	372.0	0.88 [0.47, 1.00]	0.83 [0.81, 0.86]
Costello-Boerrigter 2006c	5	65	1	326	>=65	Females	372.0	0.83 [0.36, 1.00]	0.83 [0.79, 0.87]
Abhayaratna 2006b	19	21	4	102	75-86	Females	375.0	0.83 [0.61, 0.95]	0.83 [0.75, 0.89]
Groenning 2004a	29	209	9	425	>=50	Total	414.0	0.76 [0.60, 0.89]	0.67 [0.63, 0.71]
Abhayaratna 2006f	18	53	3	539	60-86	Males	452.0	0.86 [0.64, 0.97]	0.91 [0.88, 0.93]
Costello-Boerrigter 2006i	23	84	3	637	>=65	Total	452.0	0.88 [0.70, 0.98]	0.88 [0.86, 0.91]
Abhayaratna 2006d	17	28	3	107	75-86	Males	452.0	0.85 [0.62, 0.97]	0.79 [0.71, 0.86]
Costello-Boerrigter 2006f	17	40	3	290	>=65	Males	462.0	0.85 [0.62, 0.97]	0.88 [0.84, 0.91]
Goetze 2006d	3	75	6	1418	>55	Males	626.0	0.33 [0.07, 0.70]	0.95 [0.94, 0.96]
Goetze 2006b	3	100	2	1891	>55	Females	651.0	0.60 [0.15, 0.95]	0.95 [0.94, 0.96]
Abhayaratna 2006e	6	24	0	586	60-86	Females	710.0	1.00 [0.54, 1.00]	0.96 [0.94, 0.97]
Cosin Aguilar 2003c	3	8	9	183	49-81	Total	1780.0	0.25 [0.05, 0.57]	0.96 [0.92, 0.98]

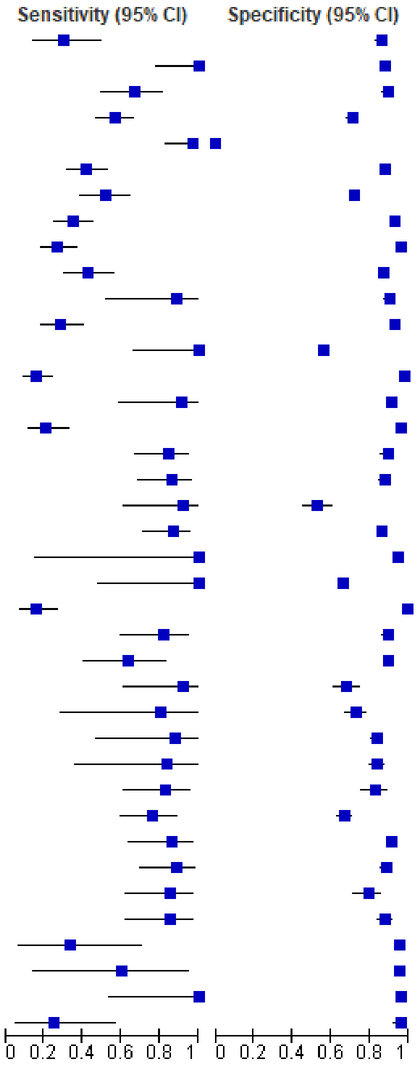

eFigure 3: Paired sensitivity and specificity plot for NP compared to echocardiography and cardiac MRI for the detection of LVSD in general screened populations

b) BNP

Study	TP	FP	FN	TN	Age group	Sex	Threshold	Sensitivity (95% CI)	Specificity (95% CI)
De Lemos 2008f	42	263	53	711	<65	Males	7.0	0.44 [0.34, 0.55]	0.73 [0.70, 0.76]
De Lemos 2008a	38	347	39	937	<65	Females	13.0	0.49 [0.38, 0.61]	0.73 [0.70, 0.75]
McDonagh 1998	30	158	10	1054	25-74	Total	17.0	0.75 [0.59, 0.87]	0.87 [0.85, 0.89]
De Lemos 2008g	21	107	75	866	<65	Males	18.0	0.22 [0.14, 0.31]	0.89 [0.87, 0.91]
Ng 2003	30	915	0	386	45-80	Total	19.2	1.00 [0.88, 1.00]	0.30 [0.27, 0.32]
Costello-Boerrigter 2006d	7	122	2	426	<65	Males	24.0	0.78 [0.40, 0.97]	0.78 [0.74, 0.81]
Vasan 2002b	39	197	21	1213	>40	Males	24.0	0.65 [0.52, 0.77]	0.86 [0.84, 0.88]
De Lemos 2008h	15	58	80	916	<65	Males	25.0	0.16 [0.09, 0.25]	0.94 [0.92, 0.95]
De Lemos 2008b	21	155	47	1136	<65	Females	26.0	0.31 [0.20, 0.43]	0.88 [0.86, 0.90]
Lukowicz 2005	4	116	1	1002	25-74	Total	27.0	0.80 [0.28, 0.99]	0.90 [0.88, 0.91]
Hedberg 2004a	26	171	2	208	75	Total	28.0	0.93 [0.76, 0.99]	0.55 [0.50, 0.60]
Vasan 2002d	8	170	2	1527	>40	Females	34.0	0.80 [0.44, 0.97]	0.90 [0.88, 0.91]
De Lemos 2008i	11	29	82	946	<65	Males	34.0	0.12 [0.06, 0.20]	0.97 [0.96, 0.98]
Luchner 2000	11	80	28	491	50-67	Total	34.0	0.28 [0.15, 0.45]	0.86 [0.83, 0.89]
De Lemos 2008c	12	78	51	1219	<65	Females	39.0	0.19 [0.10, 0.31]	0.94 [0.93, 0.95]
Costello-Boerrigter 2006g	9	202	2	909	<65	Total	42.0	0.82 [0.48, 0.98]	0.82 [0.79, 0.84]
De Lemos 2008j	7	10	84	968	<65	Males	49.0	0.08 [0.03, 0.15]	0.99 [0.98, 1.00]
Vasan 2002a	4	85	6	1612	>40	Females	50.0	0.40 [0.12, 0.74]	0.95 [0.94, 0.96]
Vasan 2003c	20	71	40	1340	>40	Males	51.0	0.33 [0.22, 0.47]	0.95 [0.94, 0.96]
De Lemos 2008d	9	39	52	1259	<65	Females	53.0	0.15 [0.07, 0.26]	0.97 [0.96, 0.98]
Costello-Boerrigter 2006e	24	155	5	723	>=50	Males	55.0	0.83 [0.64, 0.94]	0.82 [0.80, 0.85]
Smith 2000a	11	50	1	93	>70	Total	64.0	0.92 [0.62, 1.00]	0.65 [0.57, 0.73]
De Lemos 2008e	7	13	54	1286	<65	Females	65.0	0.11 [0.05, 0.22]	0.99 [0.98, 0.99]
Costello-Boerrigter 2006h	30	346	7	1486	>=50	Total	66.0	0.81 [0.65, 0.92]	0.81 [0.79, 0.83]
Smith 2000b	10	43	2	100	>70	Total	68.0	0.83 [0.52, 0.98]	0.70 [0.62, 0.77]
Hedberg 2004b	22	42	6	337	75	Total	73.0	0.79 [0.59, 0.92]	0.89 [0.85, 0.92]
Costello-Boerrigter 2006f	15	80	5	250	>=65	Males	79.0	0.75 [0.51, 0.91]	0.76 [0.71, 0.80]
Smith 2000c	9	28	3	115	>70	Total	92.0	0.75 [0.43, 0.95]	0.80 [0.73, 0.87]
Costello-Boerrigter 2006i	20	167	6	554	>=65	Total	97.0	0.77 [0.56, 0.91]	0.77 [0.74, 0.80]
Costello-Boerrigter 2006a	7	119	1	835	>=50	Females	102.0	0.88 [0.47, 1.00]	0.88 [0.85, 0.90]
Hobbs 2004	4	36	1	266	>=45	Total	103.0	0.80 [0.28, 0.99]	0.88 [0.84, 0.92]
Costello-Boerrigter 2006b	2	20	0	543	<65	Females	108.0	1.00 [0.16, 1.00]	0.96 [0.95, 0.98]
Costello-Boerrigter 2006c	5	74	1	317	>=65	Females	124.0	0.83 [0.36, 1.00]	0.81 [0.77, 0.85]

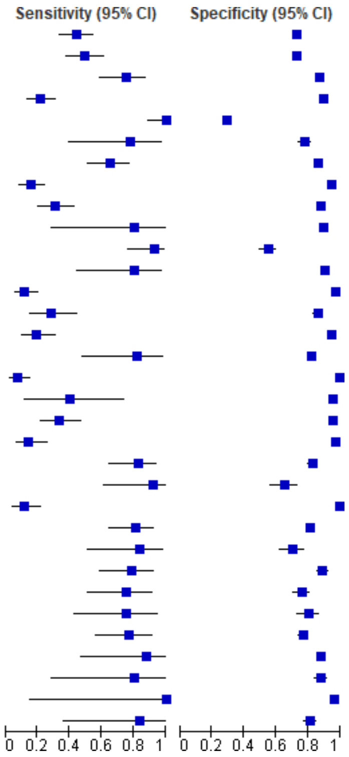

eFigure 4: SROC curve of NT-proBNP at multiple thresholds compared with echocardiography for detecting LVSD in screened general risk populations

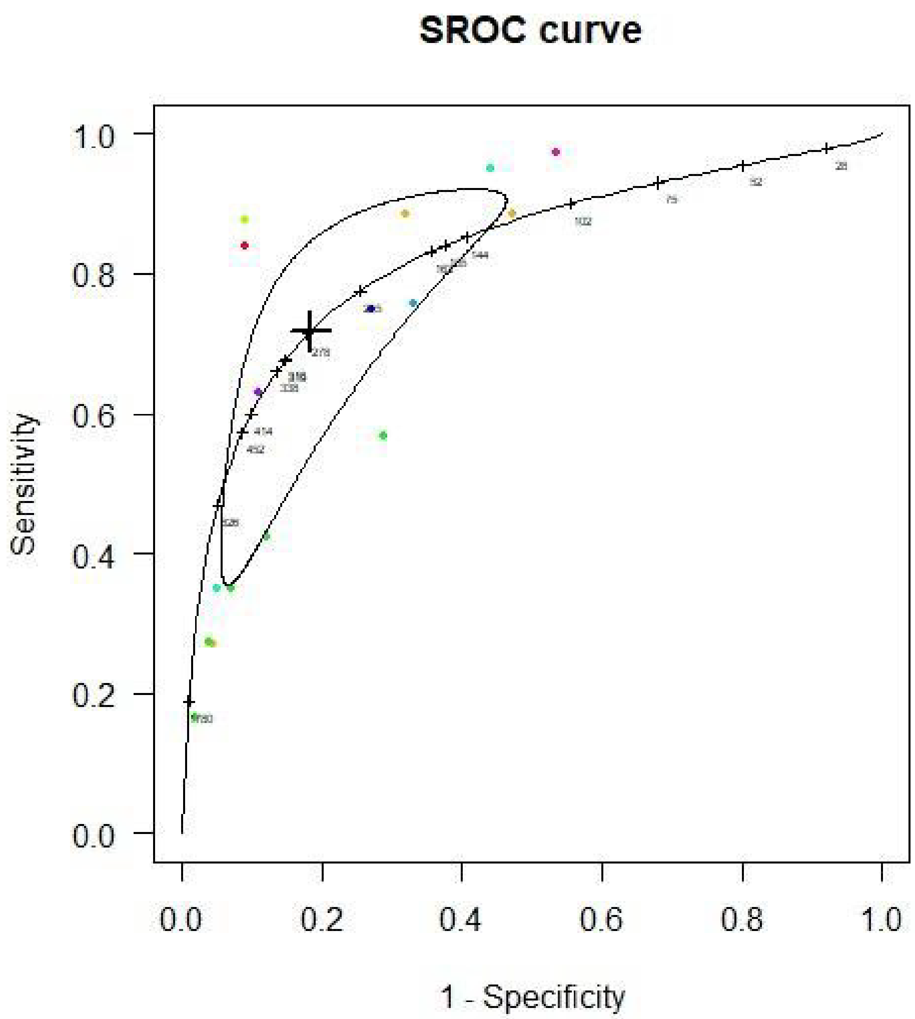

eFigure 5: SROC curve of BNP at multiple thresholds compared with echocardiography and cardiac MRI for detecting LVSD in general risk populations

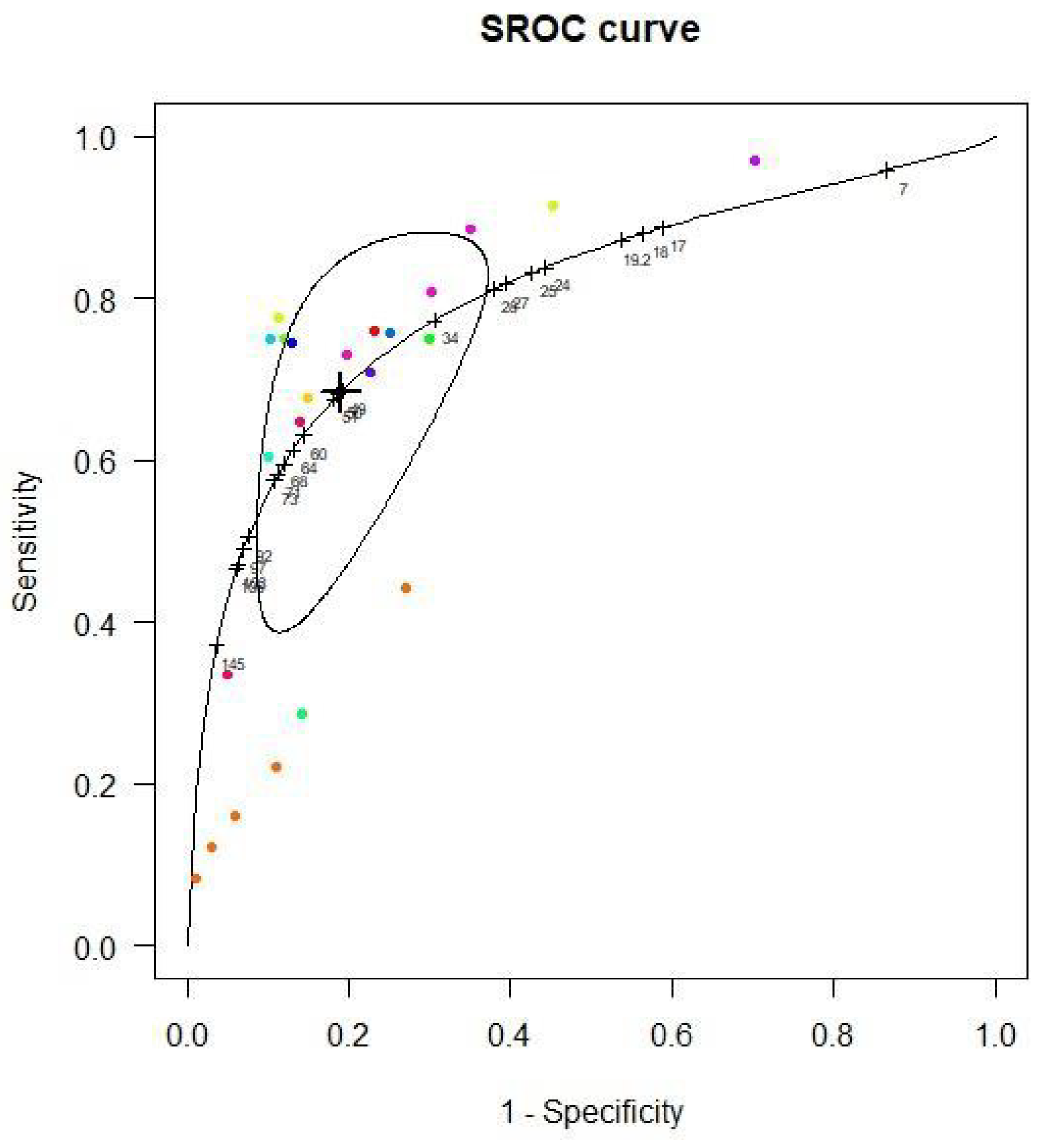

eFigure 6: SROC curve of BNP at multiple thresholds compared with echocardiography for detecting LVSD in screened general and high-risk populations combined

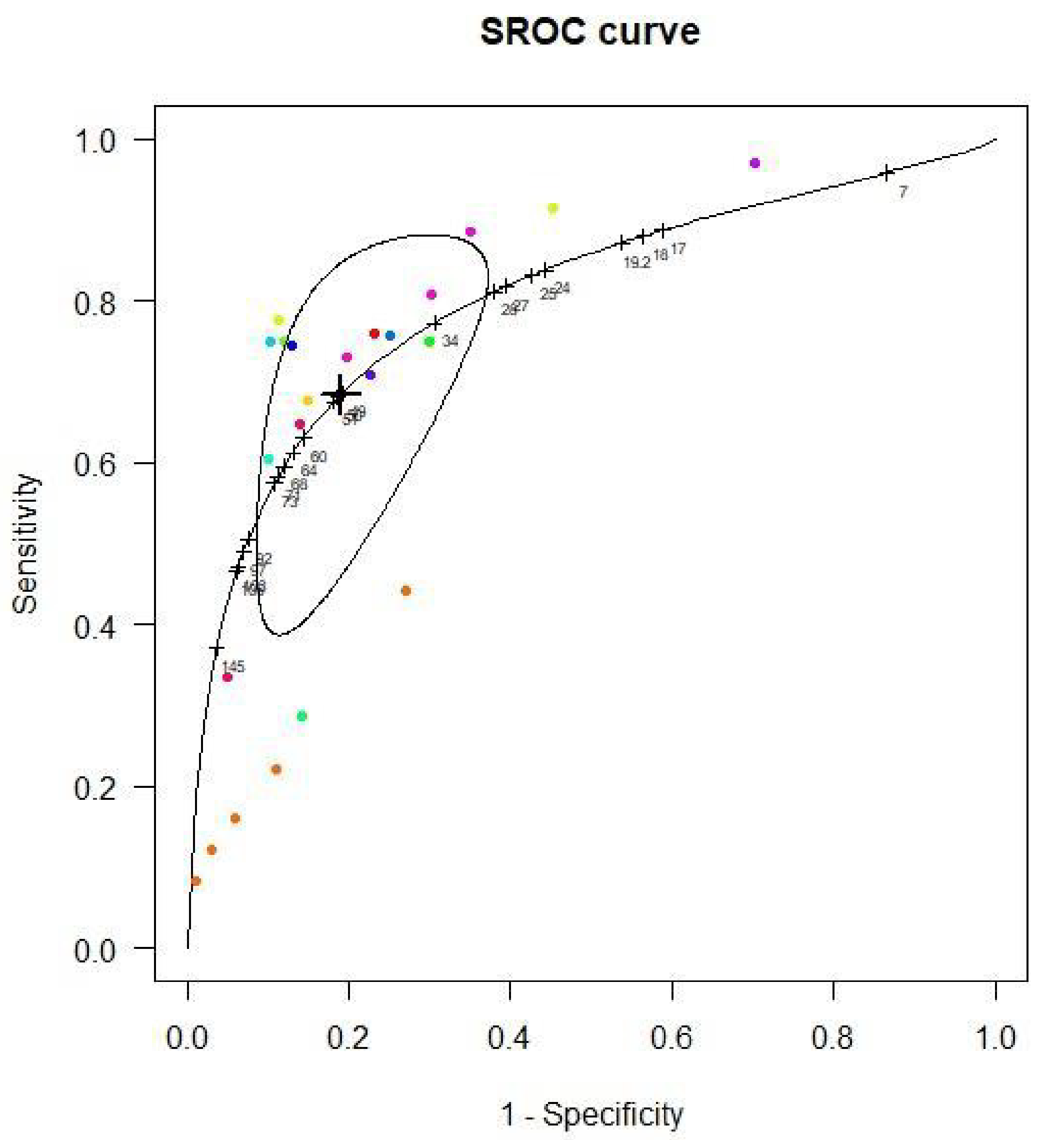

eFigure 7: Sensitivity Analysis: Paired sensitivity and specificity plot for NT-proBNP compared to echocardiography for diagnosis of LVSD in high risk screened populations with only studies that excluded patients with prior diagnosis LVSD

Study	TP	FP	FN	TN	Age group	Threshold	Sensitivity (95% CI)	Specificity (95% CI)
Luers 2010	15	62	8	457	>40	105.0	0.65 [0.43, 0.84]	0.88 [0.85, 0.91]
Goode 2007	27	219	5	176	>50	150.0	0.84 [0.67, 0.95]	0.45 [0.40, 0.50]
Hobbs 2004	10	66	0	57	>=45	338.0	1.00 [0.69, 1.00]	0.46 [0.37, 0.56]

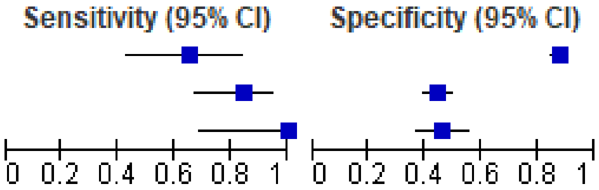

eFigure 8: Sensitivity Analysis: Paired sensitivity and specificity plot for NT-proBNP compared to echocardiography for diagnosis of LVSD in high risk screened populations with remaining studies that included screened population and did not exclude prior diagnosis LVSD specifically *Galasko thresholds based on age/sex specific 97.5th percentiles: 100pg/ml males aged 45-59; 164pg/ml females aged 45-59; 172pg/ml males aged >=60 ; 225pg/ml females age>=60. † Gavazzi thresholds based on age/sex specific 95thpercentiles (data not provided)

Study	TP	FP	FN	TN	Age group	Threshold	Sensitivity (95% CI)	Specificity (95% CI)
Galasko 2005	36	223	5	497	>=45	*	0.88 [0.74, 0.96]	0.69 [0.66, 0.72]
Mureddu 2013	6	82	0	347	65-84	278.0	1.00 [0.54, 1.00]	0.81 [0.77, 0.84]
Gavazzi 2014	6	0	3	610	55-80	†	0.67 [0.30, 0.93]	1.00 [0.99, 1.00]

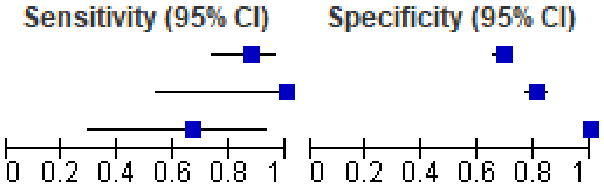

eFigure 9: Sensitivity Analysis: Paired sensitivity and specificity plot for BNP compared to echocardiography for diagnosis of LVSD in high risk screened populations with only studies that excluded patients with prior diagnosis LVSD

Study	TP	FP	FN	TN	Age group	Threshold	Sensitivity (95% CI)	Specificity (95% CI)
Murtagh 2012a	8	182	3	618	>40	50.0	0.73 [0.39, 0.94]	0.77 [0.74, 0.80]
Hebert 2010	11	19	5	110	40-75	60.0	0.69 [0.41, 0.89]	0.85 [0.78, 0.91]
Hobbs 2004	5	41	5	82	>=45	103.0	0.50 [0.19, 0.81]	0.67 [0.58, 0.75]
Luers 2010	14	52	9	467	>40	105.0	0.61 [0.39, 0.80]	0.90 [0.87, 0.92]

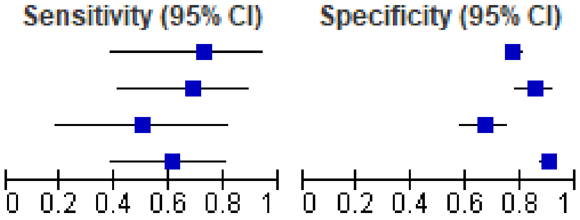

eFigure 10: Sensitivity Analysis: Paired sensitivity and specificity plot for BNP compared to echocardiography for diagnosis of LVSD in high risk screened populations with remaining studies that included screened population and did not exclude prior diagnosis LVSD specifically

Study	TP	FP	FN	TN	Age group	Threshold	Sensitivity (95% CI)	Specificity (95% CI)
McDonagh 1998	116	267	22	847	25-74	17.9	0.84 [0.77, 0.90]	0.76 [0.73, 0.79]
Lobos Bejarano 2012	13	52	4	123	>63	71.0	0.76 [0.50, 0.93]	0.70 [0.63, 0.77]

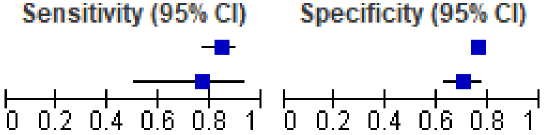

eFigure 11: Sensitivity Analysis: Paired sensitivity and specificity plot for NT-proBNP compared to echocardiography for diagnosis of LVSD in studies that described screened population as asymptomatic † Gavazzi thresholds based on age/sex specific 95thpercentiles (data not provided)

Study	TP	FP	FN	TN	Age group	Threshold	Sensitivity (95% CI)	Specificity (95% CI)
Gavazzi 2014	6	0	3	610	55-80	†	0.67 [0.30, 0.93]	1.00 [0.99, 1.00]
Luers 2010	15	62	8	457	>40	105.0	0.65 [0.43, 0.84]	0.88 [0.85, 0.91]
Betti 2009	51	192	1	768	≥67	125.0	0.98 [0.90, 1.00]	0.80 [0.77, 0.82]
Mureddu 2013	6	82	0	347	65-84	278.0	1.00 [0.54, 1.00]	0.81 [0.77, 0.84]

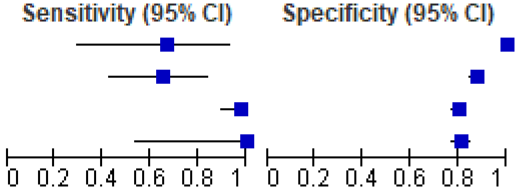

eFigure 12: Sensitivity Analysis: Paired sensitivity and specificity plot for BNP compared to echocardiography for diagnosis of LVSD in studies that described screened population as asymptomatic

Study	TP	FP	FN	TN	Age group	Threshold	Sensitivity (95% CI)	Specificity (95% CI)
McDonagh 1998	116	267	22	847	25-74	17.9	0.84 [0.77, 0.90]	0.76 [0.73, 0.79]
Murtagh 2012a	8	182	3	618	>40	50.0	0.73 [0.39, 0.94]	0.77 [0.74, 0.80]
Lobos Bejarano 2012	13	52	4	123	>63	71.0	0.76 [0.50, 0.93]	0.70 [0.63, 0.77]
Luers 2010	14	52	9	467	>40	105.0	0.61 [0.39, 0.80]	0.90 [0.87, 0.92]

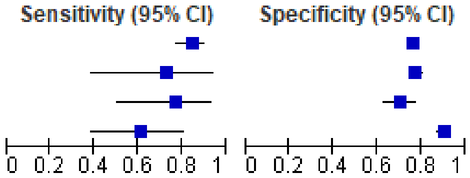

(McDonagh 1998 included as 50% symptomatic and 50% asymptomatic)

eFigure 13: Sensitivity Analysis: Paired sensitivity and specificity plot for NT-proBNP compared with echocardiography for detecting LVSD in screened high-risk populations with studies at high risk of bias (Betti 2009) removed †Gavazzi thresholds based on age/sex specific 95^{th}percentiles (data not provided) * Galasko thresholds based on age/sex specific 97.5^{th}percentiles: 100pg/ml males aged 45-59; 164pg/ml females aged 45-59; 172pg/ml males aged >=60 ; 225pg/ml females age>=60.

Study	TP	FP	FN	TN	Age group	Threshold	Sensitivity (95% CI)	Specificity (95% CI)
Gavazzi 2014	6	0	3	610	55-80	†	0.67 [0.30, 0.93]	1.00 [0.99, 1.00]
Luers 2010	15	62	8	457	>40	105.0	0.65 [0.43, 0.84]	0.88 [0.85, 0.91]
Goode 2007	27	219	5	176	>50	150.0	0.84 [0.67, 0.95]	0.45 [0.40, 0.50]
Galasko 2005	36	223	5	497	≥45	*	0.88 [0.74, 0.96]	0.69 [0.66, 0.72]
Mureddu 2013	6	82	0	347	65-84	278.0	1.00 [0.54, 1.00]	0.81 [0.77, 0.84]
Hobbs 2004	10	66	0	57	≥45	338.0	1.00 [0.69, 1.00]	0.46 [0.37, 0.56]
Mason 2013	25	86	9	273	≥65	1000.0	0.74 [0.56, 0.87]	0.76 [0.71, 0.80]

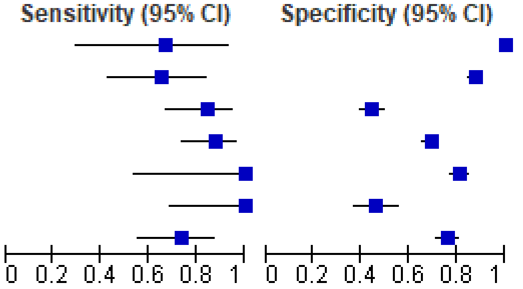

eFigure 14: Sensitivity Analysis: Paired sensitivity and specificity plot for BNP compared with echocardiography for detecting LVSD in screened high-risk populations with studies at high risk of bias (McDonagh 1998, Lobos Bejarano 2012) removed

Study	TP	FP	FN	TN	Age group	Threshold	Sensitivity (95% CI)	Specificity (95% CI)
Murtagh 2012a	8	182	3	618	>40	50.0	0.73 [0.39, 0.94]	0.77 [0.74, 0.80]
Hebert 2010	11	19	5	110	40-75	60.0	0.69 [0.41, 0.89]	0.85 [0.78, 0.91]
Hobbs 2004	5	41	5	82	>=45	103.0	0.50 [0.19, 0.81]	0.67 [0.58, 0.75]
Luers 2010	14	52	9	268	>40	105.0	0.61 [0.39, 0.80]	0.84 [0.79, 0.88]
Mason 2013	26	90	8	268	≥65	145.0	0.76 [0.59, 0.89]	0.75 [0.70, 0.79]

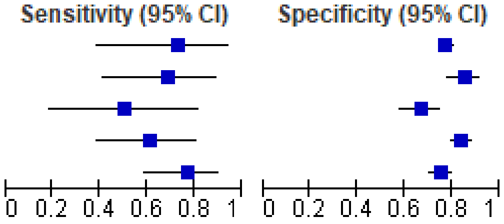

eFigure 15: Sensitivity Analysis: Paired sensitivity and specificity plot for NT-proBNP compared to echocardiography for the detection of LVSD in general screened populations with studies at high risk of bias (Costello-Boerrigter 2006, De Lemos 2008, Groening 2004) removed. * Galasko thresholds based on age/sex specific 97.5^{th} percentiles: 100pg/ml males aged 45-59; 164pg/ml females aged 45-59; 172pg/ml males aged >=60 ; 225pg/ml females age>=60.

Study	TP	FP	FN	TN	Age group	Sex	Threshold	Sensitivity (95% CI)	Specificity (95% CI)
Galasko 2005a	9	99	21	605	>=45	Total	*	0.30 [0.15, 0.49]	0.86 [0.83, 0.88]
Galasko 2005b	15	86	0	633	>=45	Total	*	1.00 [0.78, 1.00]	0.88 [0.85, 0.90]
Galasko 2005c	26	76	13	619	>=45	Total	*	0.67 [0.50, 0.81]	0.89 [0.87, 0.91]
Goetze 2006c	9	657	0	836	>55	Males	144.0	1.00 [0.66, 1.00]	0.56 [0.53, 0.59]
Abhayaratna 2006c	27	47	5	379	60-74	Males	197.0	0.84 [0.67, 0.95]	0.89 [0.86, 0.92]
Cosin Aguilar 2003a	11	90	1	101	49-81	Total	215.0	0.92 [0.62, 1.00]	0.53 [0.46, 0.60]
Goetze 2006a	5	677	0	1313	>55	Females	254.0	1.00 [0.48, 1.00]	0.66 [0.64, 0.68]
Abhayaratna 2006a	18	46	4	402	60-74	Females	270.0	0.82 [0.60, 0.95]	0.90 [0.87, 0.92]
Mureddu 2013	14	157	8	1273	65-84	Total	278.0	0.64 [0.41, 0.83]	0.89 [0.87, 0.91]
Ng 2003	29	1301	1	0	45-80	Total	318.0	0.97 [0.83, 1.00]	0.00 [0.00, 0.00]
Cosin Aguilar 2003b	11	61	1	130	49-81	Total	319.0	0.92 [0.62, 1.00]	0.68 [0.61, 0.75]
Hobbs 2004	4	82	1	220	>=45	Total	338.0	0.80 [0.28, 0.99]	0.73 [0.67, 0.78]
Abhayaratna 2006b	19	21	4	102	75-86	Females	375.0	0.83 [0.61, 0.95]	0.83 [0.75, 0.89]
Abhayaratna 2006f	18	53	3	539	60-86	Males	452.0	0.86 [0.64, 0.97]	0.91 [0.88, 0.93]
Abhayaratna 2006d	17	28	3	107	75-86	Males	452.0	0.85 [0.62, 0.97]	0.79 [0.71, 0.86]
Goetze 2006d	3	75	6	1418	>55	Males	626.0	0.33 [0.07, 0.70]	0.95 [0.94, 0.96]
Goetze 2006b	3	100	2	1891	>55	Females	651.0	0.60 [0.15, 0.95]	0.95 [0.94, 0.96]
Abhayaratna 2006e	6	24	0	586	60-86	Females	710.0	1.00 [0.54, 1.00]	0.96 [0.94, 0.97]
Cosin Aguilar 2003c	3	8	9	183	49-81	Total	1780.0	0.25 [0.05, 0.57]	0.96 [0.92, 0.98]

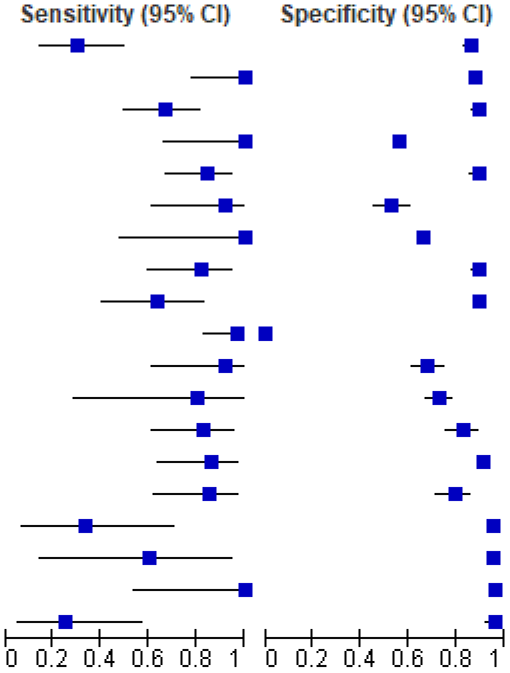

eFigure 16: Sensitivity Analysis: Paired sensitivity and specificity plot for BNP compared to echocardiography for the detection of LVSD in general screened populations with studies at high risk of bias (Costello-Boerrigter 2006, De Lemos 2008, Luchner 2000, Vasan 2002) removed

Study	TP	FP	FN	TN	Age group	Sex	Threshold	Sensitivity (95% CI)	Specificity (95% CI)
Lukowicz 2005	4	116	1	1002	25-74	Total	27.0	0.80 [0.28, 0.99]	0.90 [0.88, 0.91]
Hedberg 2004a	26	171	2	208	75	Total	28.0	0.93 [0.76, 0.99]	0.55 [0.50, 0.60]
Smith 2000a	11	50	1	93	>70	Total	64.0	0.92 [0.62, 1.00]	0.65 [0.57, 0.73]
Ng 2003	30	915	0	386	45-80	Total	66.0	1.00 [0.88, 1.00]	0.30 [0.27, 0.32]
Smith 2000b	10	43	2	100	>70	Total	68.0	0.83 [0.52, 0.98]	0.70 [0.62, 0.77]
Hedberg 2004b	22	42	6	337	75	Total	73.0	0.79 [0.59, 0.92]	0.89 [0.85, 0.92]
Smith 2000c	9	28	3	115	>70	Total	92.0	0.75 [0.43, 0.95]	0.80 [0.73, 0.87]
Hobbs 2004	4	36	1	266	>=45	Total	103.0	0.80 [0.28, 0.99]	0.88 [0.84, 0.92]

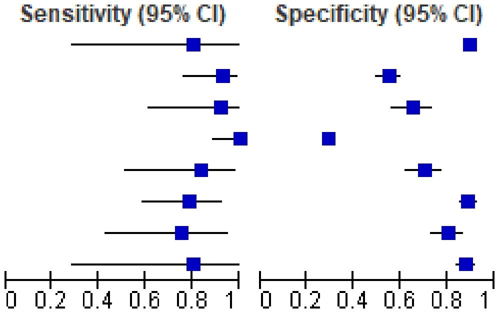

eFigure 17: Sensitivity Analysis: SROC curve of NT-proBNP at multiple thresholds compared with echocardiography cardiac MRI for detecting LVSD in general risk populations based on results for women and totals.

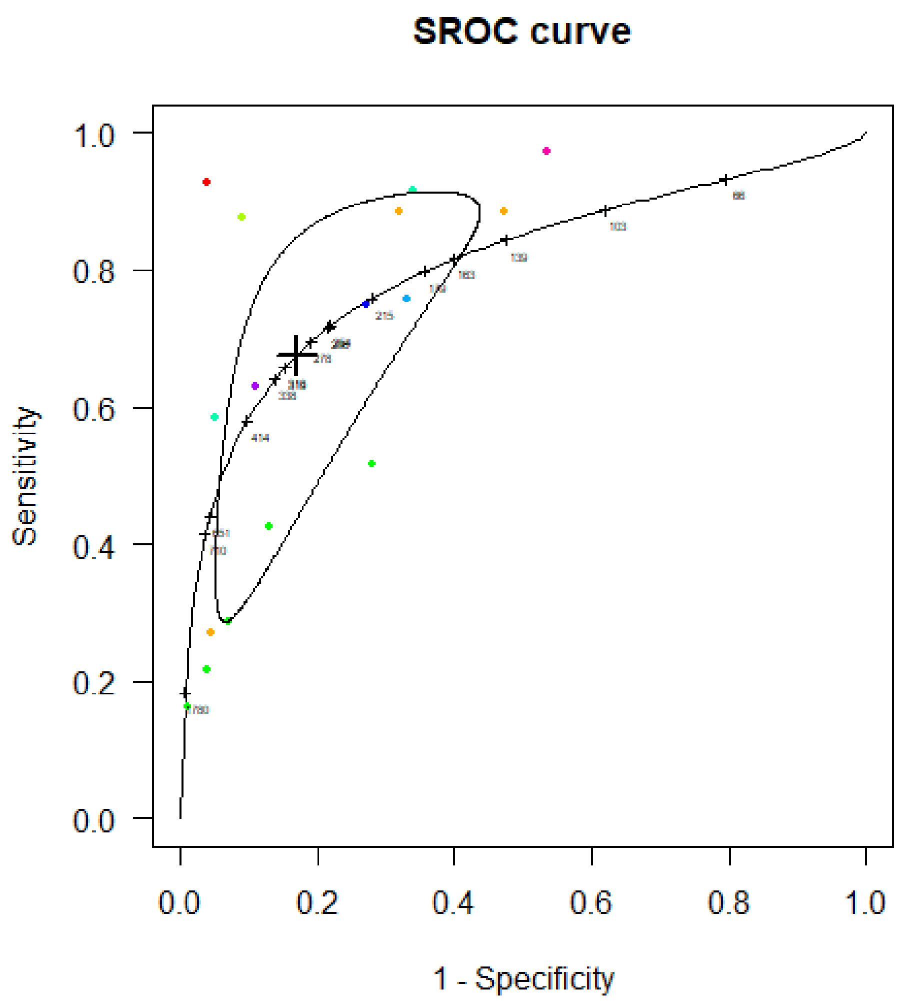

Optimal threshold 299.0 pg/ml. Sensitivity 0.68 (95% CI 0.36 – 0.89), specificity 0.83 (0.63 -0.94)

eFigure 18: Sensitivity Analysis: SROC curve of BNP at multiple thresholds compared with echocardiography cardiac MRI for detecting LVSD in general risk populations based on results for women and totals.

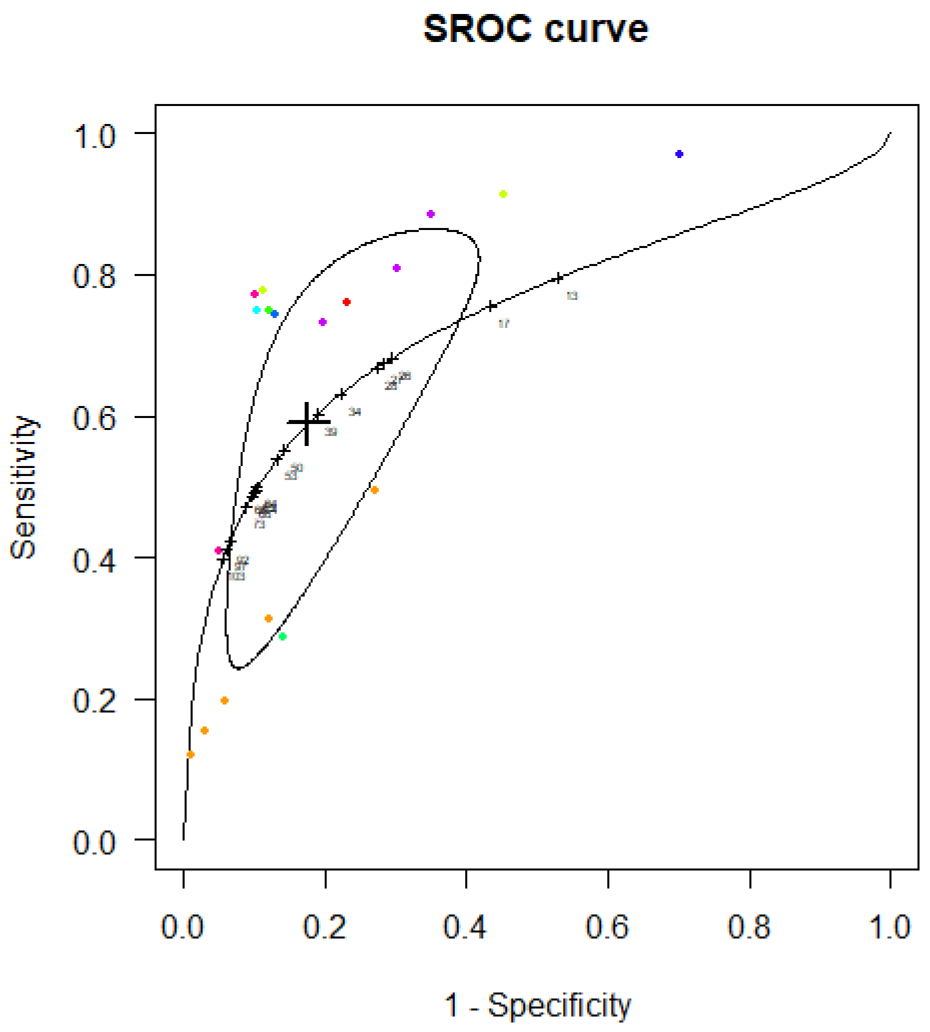

Optimal threshold 41.6 pg/ml. Sensitivity 0.59 (95% CI 0.30 – 0.83). specificity 0.82 (95% 0.64 -0.9

eFigure 19: Sensitivity Analysis : a) SROC curves of NT-proBNP compared with echocardiography for detecting LVSD in screened high-risk populations with Mason 2013 removed b) SROC curves of BNP compared with echocardiography for detecting LVSD in screened high-risk populations with Mason 2013

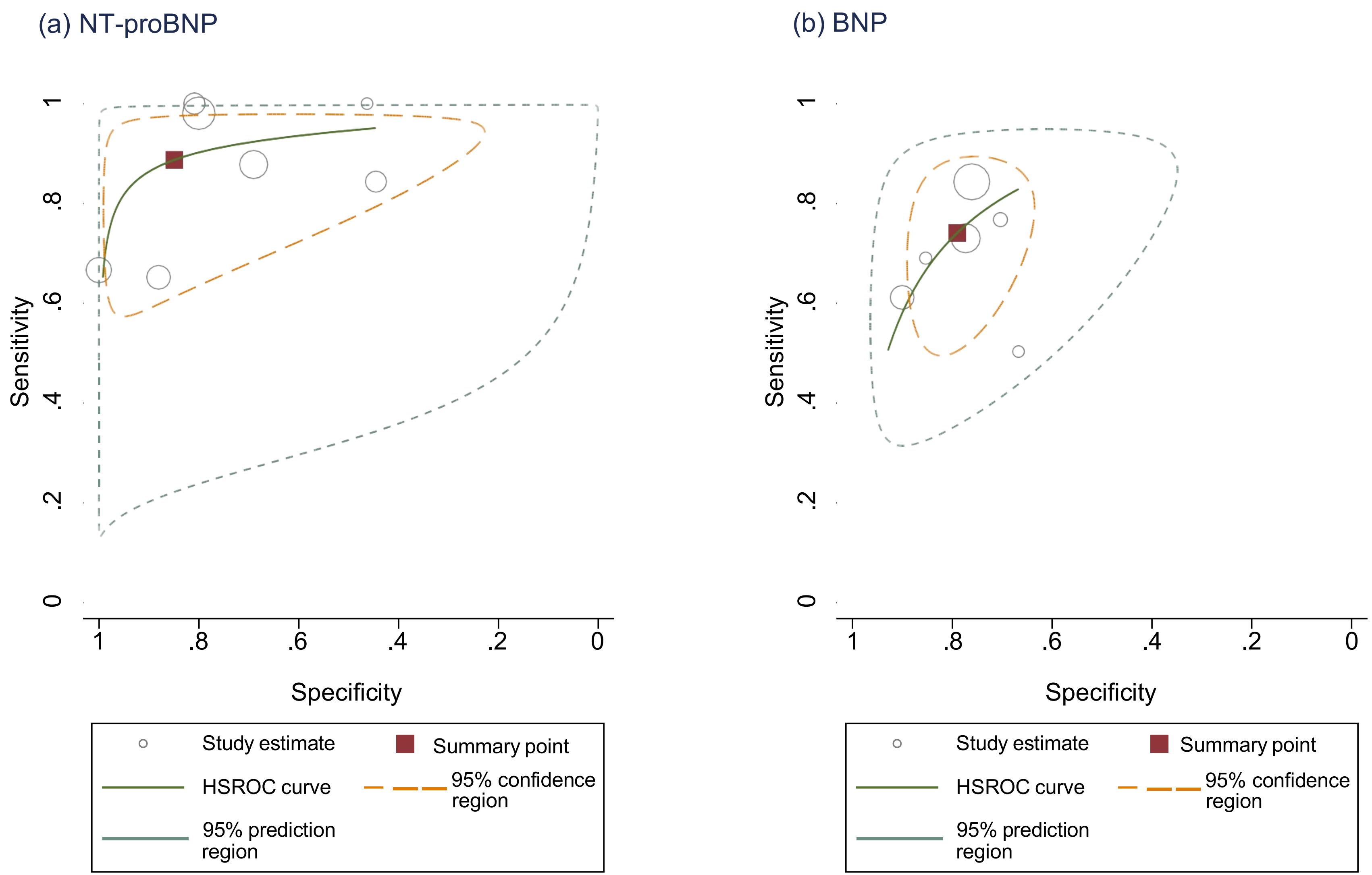

Excluding Mason 2013, for NT-proBNP, the pooled sensitivity was 0.89 (95% CI 0.74-0.96) and specificity 0.85 (95% CI 0.51 - 0.97) for detecting LVSD in screened high-risk populations. For BNP in high-risk populations the pooled sensitivity was 0.74 (95% CI 0.62 - 0.83) and specificity 0.79 (95% CI 0.71 - 0.85).
